# Environmental pH modulates transcriptomic responses in the fungus *Fusarium* sp. associated with KSHB *Euwallacea* sp. near *fornicatus*

**DOI:** 10.1186/s12864-018-5083-1

**Published:** 2018-10-01

**Authors:** Diana Sánchez-Rangel, Eric-Edmundo Hernández-Domínguez, Claudia-Anahí Pérez-Torres, Randy Ortiz-Castro, Emanuel Villafán, Benjamín Rodríguez-Haas, Alexandro Alonso-Sánchez, Abel López-Buenfil, Nayeli Carrillo-Ortiz, Lervin Hernández-Ramos, Enrique Ibarra-Laclette

**Affiliations:** 1 Red de Estudios Moleculares Avanzados, Instituto de Ecología A.C, 91070 Xalapa, Veracruz Mexico; 2 Cátedra CONACYT en el Instituto de Ecología A.C, Xalapa, Veracruz Mexico; 3 Servicio Nacional de Sanidad, Inocuidad y Calidad Agroalimentaria, Unidad Integral de Diagnóstico, Servicios y Constatación, 55740 Tecámac, Estado de México Mexico

**Keywords:** *Fusarium*, *Euwallacea*, pH, RNA-seq

## Abstract

**Background:**

The Ambrosia *Fusarium* Clade phytopathogenic *Fusarium* fungi species have a symbiotic relationship with ambrosia beetles in the genus *Euwallacea* (Coleoptera: Curculionidae). Related beetle species referred to as *Euwallacea* sp. near *fornicatus* have been spread in California, USA and are recognized as the causal agents of *Fusarium* dieback, a disease that causes mortality of many plant species. Despite the importance of this fungi, no transcriptomic resources have been generated. The datasets described here represent the first ever transcripts available for these species. We focused our study on the isolated species of *Fusarium* that is associated with one of the cryptic species referred to as Kuroshio Shot Hole Borer (KSHB) *Euwallacea* sp. near *fornicatus*.

**Results:**

Hydrogen concentration is a critical signal in fungi for growth and host colonization, the aim of this study was to evaluate the effect of different pH conditions on growth and gene expression of the fungus *Fusarium* sp. associated with KSHB. An RNA-seq approach was used to compare the gene expression of the fungus grown for 2 weeks in liquid medium at three different pH levels (5.0, 6.0, and 7.0). An unbuffered treatment was included to evaluate the capability of the fungus to change the pH of its environment and the impact in gene expression. The results showed that the fungus can grow and modulate its genetic expression at different pH conditions; however, growth was stunted in acidic pH in comparison with neutral pH. The results showed a differential expression pattern in each pH condition even when acidic conditions prevailed at the end of the experiment. After comparing transcriptomics data from the three treatments, we found a total of 4,943 unique transcripts that were differentially expressed.

**Conclusions:**

We identified transcripts related to pH signaling such as the conserved PAL/RIM pathway, some transcripts related to secondary metabolism and other transcripts that were differentially expressed. Our analysis suggests possible mechanisms involved in pathogenicity in this novel *Fusarium* species*.* This is the first report that shows transcriptomic data of this pathogen as well as the first report of genes and proteins involved in their metabolism identifying potential virulence factors.

**Electronic supplementary material:**

The online version of this article (10.1186/s12864-018-5083-1) contains supplementary material, which is available to authorized users.

## Background

Through the infection process, phytopathogenic fungi can sense and adapt to its host environment to ensure growth, reproduction, and virulence. Hydrogen ion concentration (pH) is a critical host environment variable which can limit or enhance the growth of the phytopathogenic fungi and its ability to compromise the host defense response through the secretion of pathogenicity factors such as hydrolytic enzymes [1–3] and the production of toxins [4, 5]. Because of this critical feature, some fungi have evolved to modify the host environment by actively secreting acids or alkali. For example, *Fusarium oxysporum* can alkalinize the environment by secreting ammonia [6], while the secretion of fusaric acid gives rise to extracellular acidification [4, 7, 8]. Interestingly, it has been reported that fusaric acid, through nitric oxide signaling, induces programmed cell death in their plant hosts [9]. These studies show that some pathogens have the ability to modulate environmental pH [10].

Studies have also shown that filamentous fungi have the potential to sense the concentration of hydrogen ions and respond with regulatory systems such as RIM or PAL in yeasts or filamentous fungi, respectively [11]. PAL proteins sense alkaline pH and transmit this external stimulus in two consecutive steps that produce proteolytic cleavage of a zinc finger transcription factor named PacC in filamentous fungi and RIM101 in yeasts [11]. The active PacC is translocated to the nucleus where it can recognize the consensus DNA site 5’-GCCAAG-3′ and consequently up- or down-regulate the transcription of acidic- or alkaline-pH responsive genes, respectively [12, 13]. Some pathogenic factors regulated by the Pal/Rim system in fungi include: (*i*) pectate lyase (pelB) in *Colletotrichum gloeosporioides* [3], (*ii*) endoglucanases in *Alternaria alternata* [1], (*iii*) synthesis of extracellular polysaccharides and oxalic acid as well as production of polygalacturonase and laccase enzymes *in Botrytis cinerea* [14] and others [15].

*Fusarium* is a cosmopolitan genus that includes species of necrotrophic fungi that cause serious phytosanitary problems, and there is evidence that some relevant virulence factors are regulated by external pH, both in a PacC-dependent and -independent manner. For example, the homolog of PacC in *Fusarium graminearum*, *FgPac1*, regulates *Tri* gene cluster expression and the trichothecene production [16]. In *Fusarium verticillioides*, the fumonisin production is enhanced in acidic pH and is negatively regulated by the PacC homolog [17]. In addition, two relevant endo-polygalacturonase (*pg1* and *pg5*) are downregulated by a mechanism independent of PacC in *F. oxysporum* at alkaline pH [4].

Recently, two *Fusarium* species belonging to the Ambrosia *Fusarium* Clade (AFC) have been discovered. These species have a symbiotic relationship with cryptic species of the Asian beetle referred to as *Euwallacea* sp. near *fornicatus* and have been spread in California, USA. Moreover, they have been recognized as the causal agents of *Fusarium* dieback in forests, landscape trees, and in the agricultural sector, causing significant losses in the avocado industry [18, 19]. *Fusarium euwallaceae* is known to be associated with the Polyphagous Shot Hole Borer (PSHB) [20] and *Fusarium sp.* is associated with the Kuroshio Shot Hole Borer (KSHB) that recently has been named *F. kuroshium* sp. [21]. In 2016, KSHB was also discovered in Baja California, Mexico [22], and despite the considerable interest in *Fusarium* species associated with ambrosia beetles, no transcriptomic data are available to help understand the nature of these pathogens, as well as the virulence factors required for disease establishment and development. In this sense, an effective strategy to evaluate the effect of pH on the behavior of a fungus is to challenge it at different pH levels and to analyze their effect on conidiation, radial growth, or fresh weight [23], to identify specific responses at the gene level [24], as well as to evaluate large-scale responses [25].

In this study, we evaluated the ability of this fungus to growth at different pH levels and analyzed the associated transcriptomic responses. We used the *Fusarium* sp. associated with KSHB (strain HFEW-16-IV-019) and cultured it at distinct pH conditions (5.0, 6.0, and 7.0) for 2 weeks. Following that, we used an RNA-seq approach to identify pH-responsive genes including the signaling route Pal/Rim. In addition, we identified some known elements (genes/proteins) in the fungi virulence arsenal and evaluated the expression profile at different pH levels.

Besides, those nr-unigenes that showed the most notable change and homologous to those genes involved in the biosynthesis of polyketide-derived mycotoxins were identified. Additionally, to the canonical genes, we were able to identify some nr-unigenes related with pathogenicity that for the first time has been recognized in this fungus. The innovation of these data implies the first transcriptomic knowledge of this important phytopathogen that is a symbiont of the beetle *Euwallacea* sp. near *fornicatus*, giving interesting clues about the molecular mechanisms that govern its growth and adaptation to abiotic stress. Finally, this is the first report that documents the ability of this fungus to grow in different pH conditions and some transcriptomic responses associated with this adaptation.

## Results

### Effect of pH on conidiation and growth of *Fusarium* sp. associated with KSHB

**Fig. 1:**
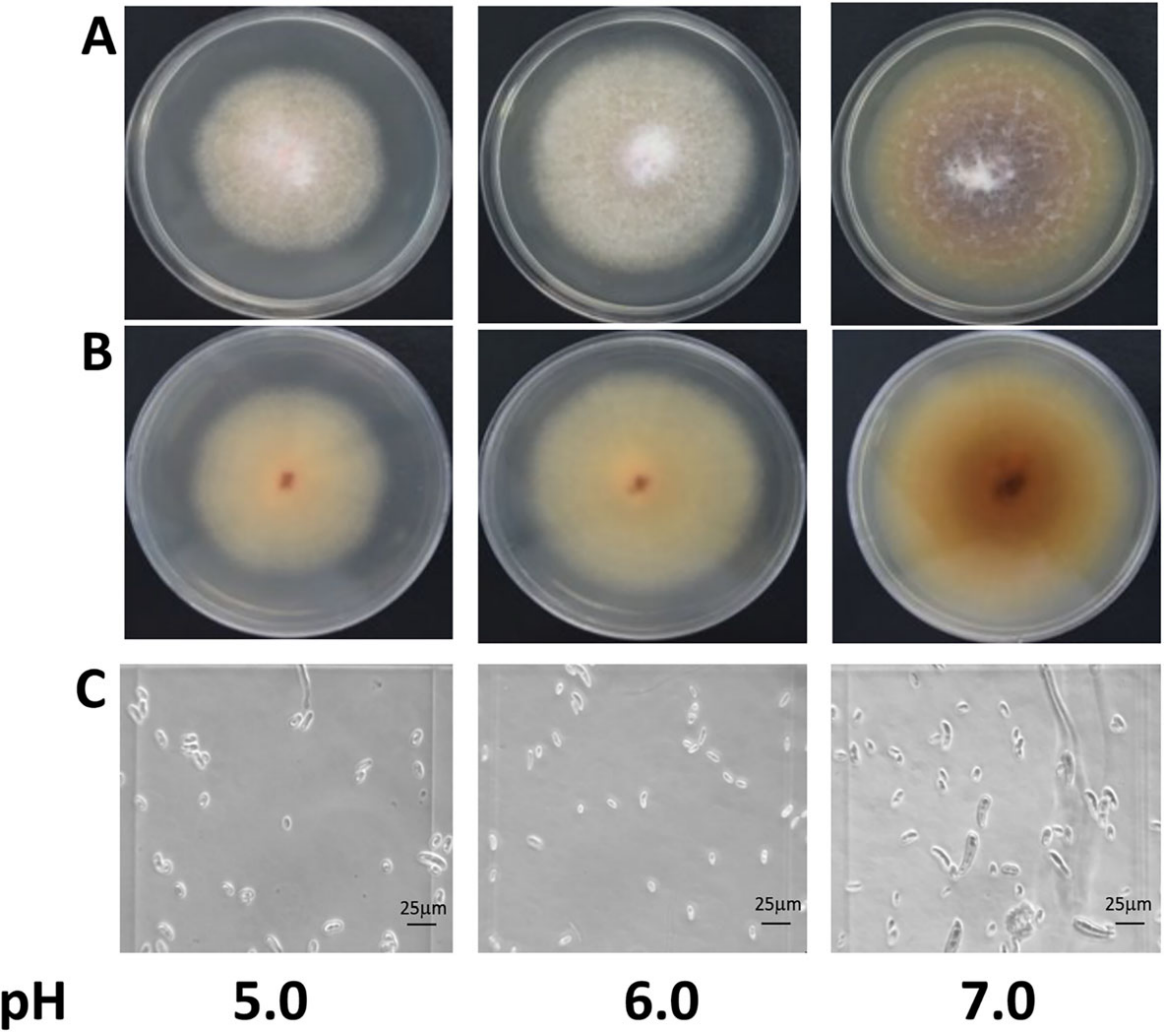
Effect of pH on the growth and conidiation of *Fusarium* sp. associated with KSBH. Macroscopic morphology of *Fusarium* sp. associated with KSBH growing in PDA for 9 days at 29 °C in dark conditions as seen from the obverse (**a**) and reverse (**b**) side of the plates. Microscopic conidia morphology was observed after they were harvested from PDA plates and re-suspended in distilled water (**c**)

To characterize the influence of pH on fungal conidiation and growth, *Fusarium* sp. associated with KSHB was grown in liquid media (PDB) and microscopic observations were recorded, and fresh weight was measured. The same strain was grown on solid (PDA) culture media, and the radial growth and the colony phenotype differences were observed. After 9 days of incubation at 28 ± 2 °C in complete darkness, *Fusarium* sp. associated with KSHB showed markedly reduced radial growth at pH 5.0 in comparison with pH 6.0 and 7.0, whereas pH 7.0 is the condition with the highest observed colony diameter (Fig. 1). Colony color on PDA plates (obverse view) was white and pale yellow at pH 5.0 and 6.0, while at pH 7.0, the color was purplish-gray (Fig. 1a). The reverse view of the plates showed a colony-pigmentation that ranged from pale yellow (pH 5.0 and 6.0) to brownish orange (pH 7.0) (Fig. 1b).

The mycelial growth in liquid media at different pH levels was also evaluated by recording differences in fresh weight. The highest growth was at pH 7.0 (13.09 ± 0.49 g), whereas it was intermediate at pH 6.0 (10.69 ± 0.53 g) and was the lowest at pH 5.0 (8.29 ± 0.16 g). At the end of the experiment (2 weeks post-inoculation), the pH of the liquid medium was measured. Interestingly, regardless of the initial pH condition, in all the cases the pH was more acidic than the initial condition. The final values of the initial pH 5.0, 6.0, and 7.0 conditions were 3.8 ± 0.04, 4.35 ± 0.14, and 4.84 ± 0.20, respectively. The conidia analysis indicated that at pH 7.0 the production of the particular conidia of this species was induced (a dolphin-like appearance; [20]). These kinds of conidia are swollen in their upper half commonly with 4 or 5 septa. The conidia at pH 5.0 and 6.0 showed no differences and were small with 1 or 2 septa, and no dolphin-like conidia were detected (Fig. 1c).

### Construction of the unigenes set for *Fusarium* sp. associated with KSHB species

A total of 151,009,935 paired-end reads were generated from nine distinct libraries that represent each of the evaluated conditions (pH 5.0, 6.0, and 7.0) which were sequenced in triplicate. On average, each of the three sequenced replicates contributed approximately 17 million paired reads. Before de novo assembly, raw reads were filtered to only include those with acceptable quality (Additional file 1: Table S1; see Methods for more details). Longer reads were generated by merging paired-end reads with overlapping regions. Both data sets (long reads and no merged paired-end reads) were used to assemble the transcriptome of *Fusarium* sp. associated with KSHB (Additional file 1: Table S1). A total of 37,550 unigenes were generated ranging from 200 to 24,657 bp, with an average length of 1,900.81 bp (Additional file 2).

Out-of-frame insertions/deletions in coding regions were corrected using the AlingWise pipeline [26] which correct the erroneous frame shifts through a homology-based method (see Methods section for more details). After correcting the frame-shifts, redundant sequences were eliminated. We considered a sequence redundant if presented an identity of at least 95% over 90% or more of the length of the sequences against which it was compared. The corrected and non-redundant (nr) unigenes set comprises a curated transcripts collection of 25,070 sequences representative of the *Fusarium* sp. associated with KSHB transcriptome with identified and translated open reading frames (ORFs) ranging between 15 to 5,692 amino acids (Additional file 1: Table S2). Only these sequences were considered for future analysis.

### Homology search and functional annotation of *Fusarium* sp. associated with KSHB unigenes

The annotation process for nr-unigenes included a functional classification and a search for similar sequences in sets of predicted proteins of the filamentous fungi whose genomes have been completely sequenced and that we considered closely related species or representatives of specific classes. The species included in the comparisons which were conducted using the Basic Local Alignment Search Tool (BLAST; [27]) were: *Botrytis cinerea* [28, 29] [Class: Leotiomycetes]; *Symbiotaphrina kochii* [Class: Xylonomycetes]; *Acremonium alcalophilum, Fusarium fujikuroi* [30]*, Fusarium graminearum* [31]*, Fusarium oxysporum* [32]*, Nectria haematococca* ([33]; commonly referred to by its asexual name *Fusarium solani*), *Fusarium verticillioides* [31, 32]*, Neurospora crassa* [34] and *Verticillium dahliae* [35]. With only a few exceptions (*S. kochii, A. alcalophilum,* and *N. crassa*), most of the selected fungi are considered highly pathogenic in a wide range of crops. Species such as *S. kochii* were included because *Symbiotaphrina* is a yeast-like genus of endosymbionts of beetles which have been suggested to be implicated in B-vitamin biosynthesis, fatty acid metabolism, and detoxification of noxious plant compounds by insects [36]. On the other hand, *A. alcalophilum* is a cellulolytic fungus whose optimal growth depends on alkaline conditions [37]. *N. crassa* was included because it is the model system for the understanding of light responses in fungi [38]. Also, *Saccharomyces cerevisiae* [Class: Saccharomycetes]; was included because it has the best-annotated genome from the Ascomycota phylum [39, 40].

**Fig. 2:**
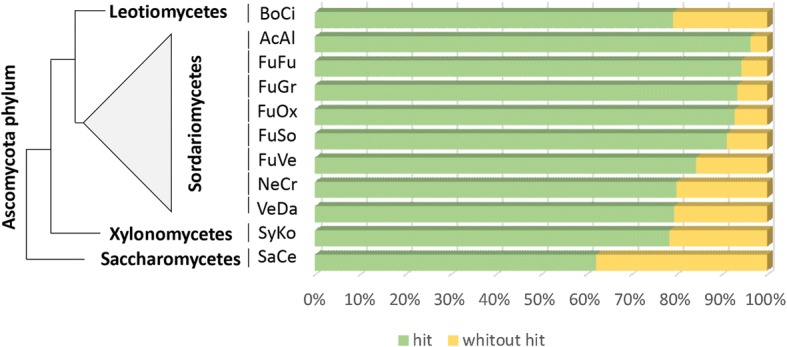
Percent of annotated nr-unigenes with or without hits. BLASTp similarity searches were performed using the translated CDS identified in *Fusarium* sp. associated with KSBH nr-unigenes against the proteins reported from some fungi species with sequenced whole genomes: *Botrytis cinerea* (BoCi)*, Symbiotaphrina kochii* (SyKo)*, Acremonium alcalophilum* (AcAl)*, Fusarium fujikuroi* (FuFu)*, Fusarium graminearum* (FuGr)*, Fusarium oxysporum* (FuOx)*, Nectria haematococca* (commonly referred to by its asexual name *Fusarium solani* (FuSo)), *Fusarium verticillioides* (FuVe)*, Neurospora crassa* (NeCr) and *Verticillium dahliae* (VeDa). The *Saccharomyces cerevisiae* (SaCe) yeast, was also included. The number of homologs identified from each species was different, and larger numbers were found in closely related species

As expected, once unigenes were corrected and filtered, most of the proteins produced by translated the ORFs, showed high similarity (e-value < 10^− 5^) with proteins from at least one of the ten species against which they were compared (Additional file 1: Table S2). In more than 90% of the *Fusarium* sp. associated with KSBH nr-unigenes set, homologous proteins were detected from other species of *Fusarium* genus. In minor proportion and decreasing order, homologous proteins were detected from *V. dahliae*, *N. crassa*, *A. alcalophilum* belonging to Sordariomycetes, *B. cinerea* belonging to Leotiomycetes, *S. kochii* belonging to Xylonomycetes, and *S. cerevisiae* belonging to Saccharomycetes (Fig. 2). *Fusarium* sp. associated with KSBH proteins produced by translated ORFs were classified as ‘complete’ or ‘partial’. Proteins were considered complete if they covered at least 70% the length of four or more identified homologous proteins. Based on this criterion, 9,392 nr-unigenes (37.5% of the total), may represent full-length cDNAs from *Fusarium* sp. associated with KSBH (see Additional file 1: Table S2).

**Fig. 3:**
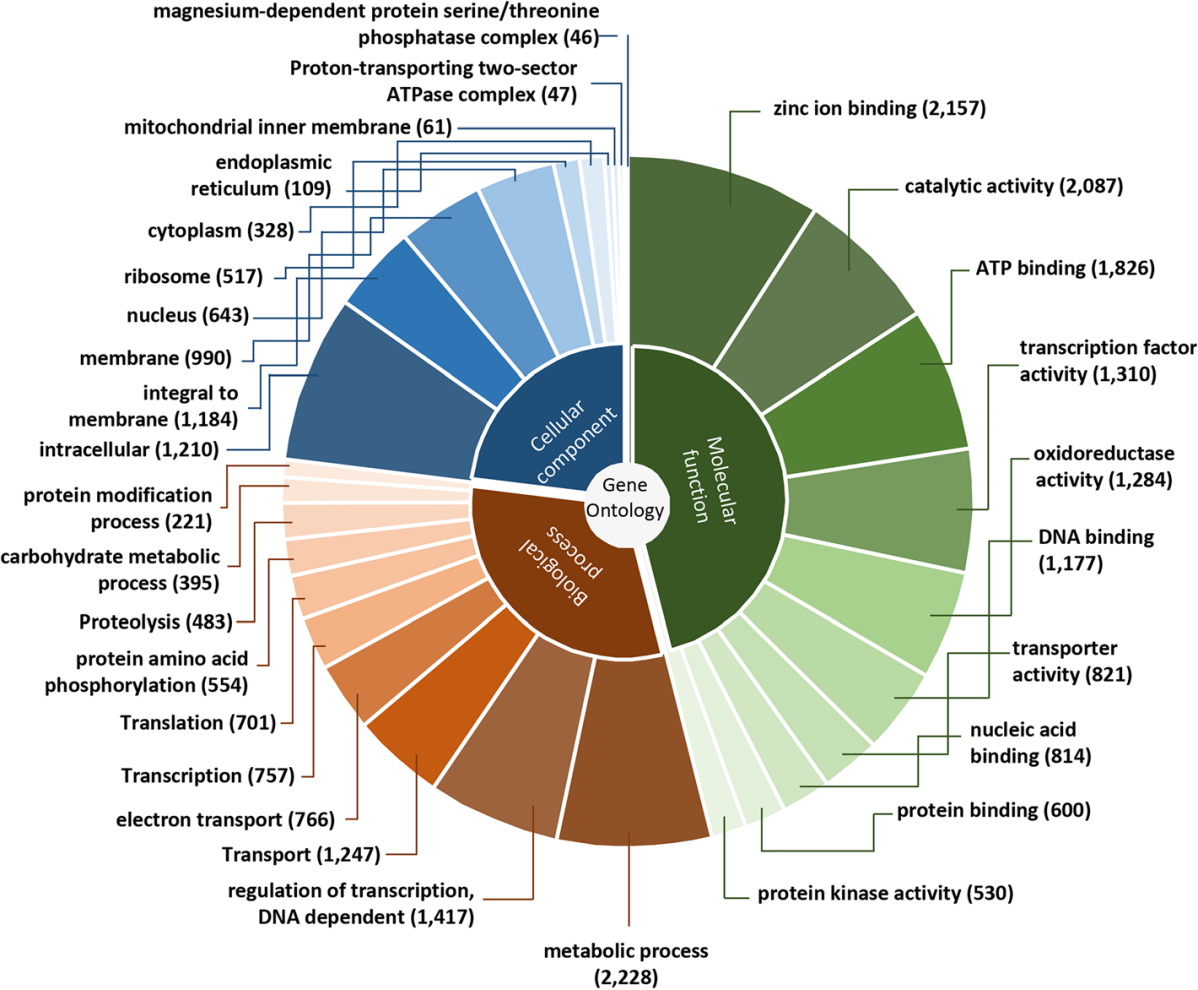
Gene ontology functional characterization of *Fusarium* sp. associated with KSBH unigenes. The results are summarized in three major categories as Molecular Function, Biological Process, and Cellular Component. Slices show top ten most represented GO terms from each category. The number in brackets refers to the total number of GO terms which have been assigned to each category

Gene ontology (GO) terms [41], enzyme commission (EC) number [42], the KOG identification number (from Eukaryotic Orthologous Groups of proteins [43]) and the most representative InterPro (IPR) domains [44–46], were assigned to each nr-unigenes based on the information available from the homologous detected (Additional file 3: Table S3). Of the three major GO annotation categories, molecular functions comprised 66.52% of the total assigned annotations, whereas biological processes and cellular components comprised 22.50% and 10.99%, respectively. The GO terms with the largest number of assigned sequences in the molecular function category were zinc ion binding (2,157 nr-unigenes), catalytic activity (2,087), ATP binding (1,826), and transcription factor activity (1,310). Out of the biological processes, the terms with the most sequences were metabolic processes (2,228), regulation of transcription/DNA-dependent (1,417), transport (1,247), and electron transport (766). In the cellular components’ category, the terms with the most sequences were intracellular (1,210), integral to membrane (1,184), membrane (990), and nucleus (643). Figure 3 shows the top ten most represented GO terms from each of the three major categories: molecular functions, biological processes, and cellular components.

### Metabolic pathway assignments

Regarding the representation or coverage of metabolic pathways represented in *Fusarium* sp. associated with KSBH transcriptome, EC numbers assigned to nr-unigenes were used to generate a metabolic network for this species. This *Fusarium* sp. associated with KSBH metabolic network was compared against a background metabolic network which was generated combining the total of EC numbers assigned to each of the proteins presents in the genomes of distinct species of the genus *Fusarium* (*F. fujikuroi, F. graminearum, F. oxysporum, F. solani* and *F. verticillioides*). Metabolic networks were reconstructed using “Search & Color Pathway” tool (http://www.genome.jp/kegg/tool/map_pathway2.html) available in the tools suite of KEGG database [47, 48]. The background network of the *Fusarium* genus comprises a total of 115 metabolic pathways in which 4,174 EC numbers were assigned. Although the metabolic network of *Fusarium* sp. associated with KSBH shows the same number of metabolic pathways to those present in the *Fusarium* background metabolic network, these pathways are represented by slightly fewer numbers of enzymes (3,372 EC numbers, 80% of the total; Additional file 4: Figure S1 and Additional file 3: Table S4). These data suggest that the presented transcriptome comprises a deep coverage of the protein coding-genes present in the genome of *Fusarium* sp. associated with KSBH.

### Identification of orthologous groups across *Fusarium* species

OrthoMCL is an analysis pipeline that uses the reciprocal BLAST and the Markov CLuster (MCL) algorithm to infer and group orthologs (and paralogs) across multiple taxa (see Methods for more details). In addition to homolog searches presented and discussed before, an orthoMCL clustering analysis was performed to identify and group (putative) orthologs across *Fusarium* species. *S. cerevisiae* was also included in the OrthoMCL analysis because it remains the best available annotated genome in the Ascomycota phylum. A total of 17,908 ortholog groups were identified among compared species (Additional file 3: Table S5). These OrthoMCL-defined protein families group a total of 91,680 proteins (~ 82% of the total included in the analysis). 17,791 of *Fusarium* sp. associated with KSBH nr-unigenes (70.96% of the total), were assigned to at least one these groups. In total, 6,424 proteins (grouped in a total of 6,205 families) were common among all *Fusarium* species (Additional file 4: Figure S2 and Additional file 3: Table S5). The ortholog identification made possible the non-erroneous assignation of the functions and expression profiles to each of the nr-unigenes that were analyzed and discussed in sections described below.

### Phylogenetic relationship with additional strains of the Ambrosia *Fusarium* Clade

*Fusarium* species that maintain mutualistic association with ambrosia beetles are grouped in the Ambrosia *Fusarium* Clade (AFC), a strongly supported monophyletic group composed of numbered species without a name [19, 49]. This monophyletic group (AFC) was resolved within the *F. solani* species complex (FSSC) using a four-locus phylogenetic analysis. The locus included in the analysis were: (*i*) a portion of the nuclear ribosomal RNA gene repeat (rDNA) comprising the internal transcribed spacer region (ITS) together with domains D1 and D2 of the nuclear ribosomal large subunit (LSU, 1,004 bp total alignment); (*ii*) translation elongation factor 1 alpha (EF-1α, 687 bp alignment); (*iii*) DNA-directed RNA polymerase II subunit 1 (RPB1, 1,588 bp alignment) and (*iv*) DNA-directed RNA polymerase subunit 2 (RPB2, 1,635 bp alignment) [49]. To infer the phylogenetic relationship of *Fusarium* sp. associated with KSBH, strain HFEW-16-IV-019, which was used in the presented work, we first identify the corresponding nr-unigenes to those homologous used in the previously reported four-locus analysis [21, 49]. With only one exception (EF-1α, UN25284), the identified nr-unigenes represent complete coding sequences from each of the locus mentioned above (UN01385 for RB1, UN04406 for RB2, and UN02180 for LSU). The four loci were concatenated into a single matrix (4,703 bp aligned) and maximum likelihood phylogenetic tree (Additional file 4: Figure S3) was generated for a total of 49 sequences which were alignment (Additional file 5). The strain HFEW-16-IV-019 was brought together with the two sister clades which group the isolates of *Fusarium* sp. previously designated to particular genealogical lineage named AF-12. The later lineages represent *Fusarium* species that come from *Euwallacea* sp. and were isolated either from the beetle or tree galleries of the host species identified in California, USA. The strain HFEW-16-IV-019 recently included in the four-locus analysis and isolated from beetles or elm (*Ulmus* sp.) tree galleries from Tijuana, Baja California, Mexico was not only grouped with the AF-12 lineage but also with AF-13 lineage, which is represented by *Fusarium* species isolated from the *Euwallacea* sp. from Taiwan (Additional file 4: Figure S3).

### Differential massive expression analysis of pH-responsive genes in *Fusarium* sp. associated with KSBH

To compare the expression levels of the nr-unigenes among samples (pH 5.0, 6.0 and 7.0) and identify those which are differentially expressed, sequences count-based differential expression analysis was performed. RSEM [50] and DEseq [51] were the programs used for these purposes. First, an expression profile matrix was created containing the number of expected reads for each of the 25,070 nr-unigenes (rows) in each of conditions analyzed (columns). Also, the normalized values TPM (transcripts per million) and FPKM (fragments per Kilobases of contigs/unigenes for per million mapped reads) were calculated (Additional file 3: Table S6). Considering that recently it has been reported that FPKM is an inconsistent measure to compare among samples [52, 53], in this work the TPM values were chosen as the representative value of the expression profiles. Differentially expressed genes were identified comparing the expected read counts normalized by the DEseq package between each pair of samples. In total, 4,785 genes (Additional file 3: Table S7) were identified as differentially expressed in at least one of the three comparisons that were performed (pH 5.0/pH 6.0, pH 5.0/pH 7.0, and pH 7.0/pH 6.0).

**Fig. 4:**
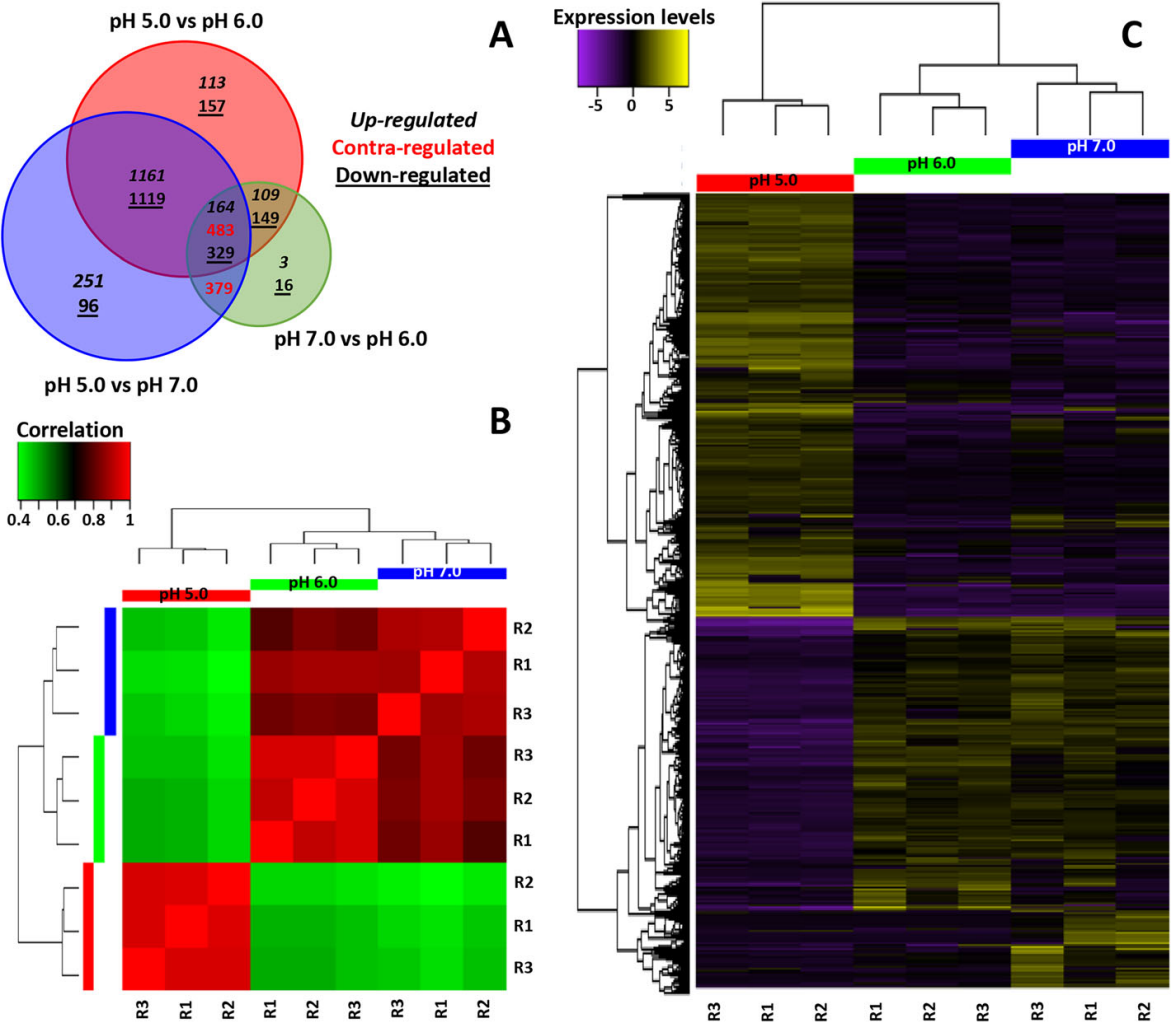
Genes differentially expressed under distinct pH conditions. **a** Venn diagram shows the amount of differentially expressed genes, common or specific to the pairwise compared samples. **b** Heat map which indicates the correlation between transcriptional profiles across samples (Spearman correlation). In this case, the color scale indicates the degree of correlation between the treatments (pH 5.0, 6.0, and 7.0). Green and red represent a weak or strong correlation between the samples, respectively. **c** The expression profile of pH-responsive genes showing as a heat map after DEseq normalization and the hierarchical clustering analysis. TPM (transcripts per million) values in a log_2_ scale were used. These values were independently calculated for each of the three biological replicates (R1, R2, and R3) performed for each of the three conditions (pH 5.0, 6.0, and 7.0, respectively). For coloring purposes, each row represents the expression of a single transcript (nr-unigenes) where shades of yellow represent high expression levels while the shades of purple represent low expression levels. In both heat maps, biological replicates are shown independently to show their reproducibility

The Venn diagrams generated with the differentially expressed genes show that most of these genes are common between pH 5.0/pH 6.0 and pH 5.0/pH 7.0 comparisons (Fig. 4a). On the other hand, only a few differentially expressed genes are identified between the pH 7.0 and pH 6.0 treatments. Hierarchical clustering and heatmap analyses show similar expression patterns with subtle changes between the pH 6.0 or pH 7.0 treatments. Interestingly, both treatments show significant differences when they are compared with pH 5.0 (Fig. 4b and c).

**Fig. 5:**
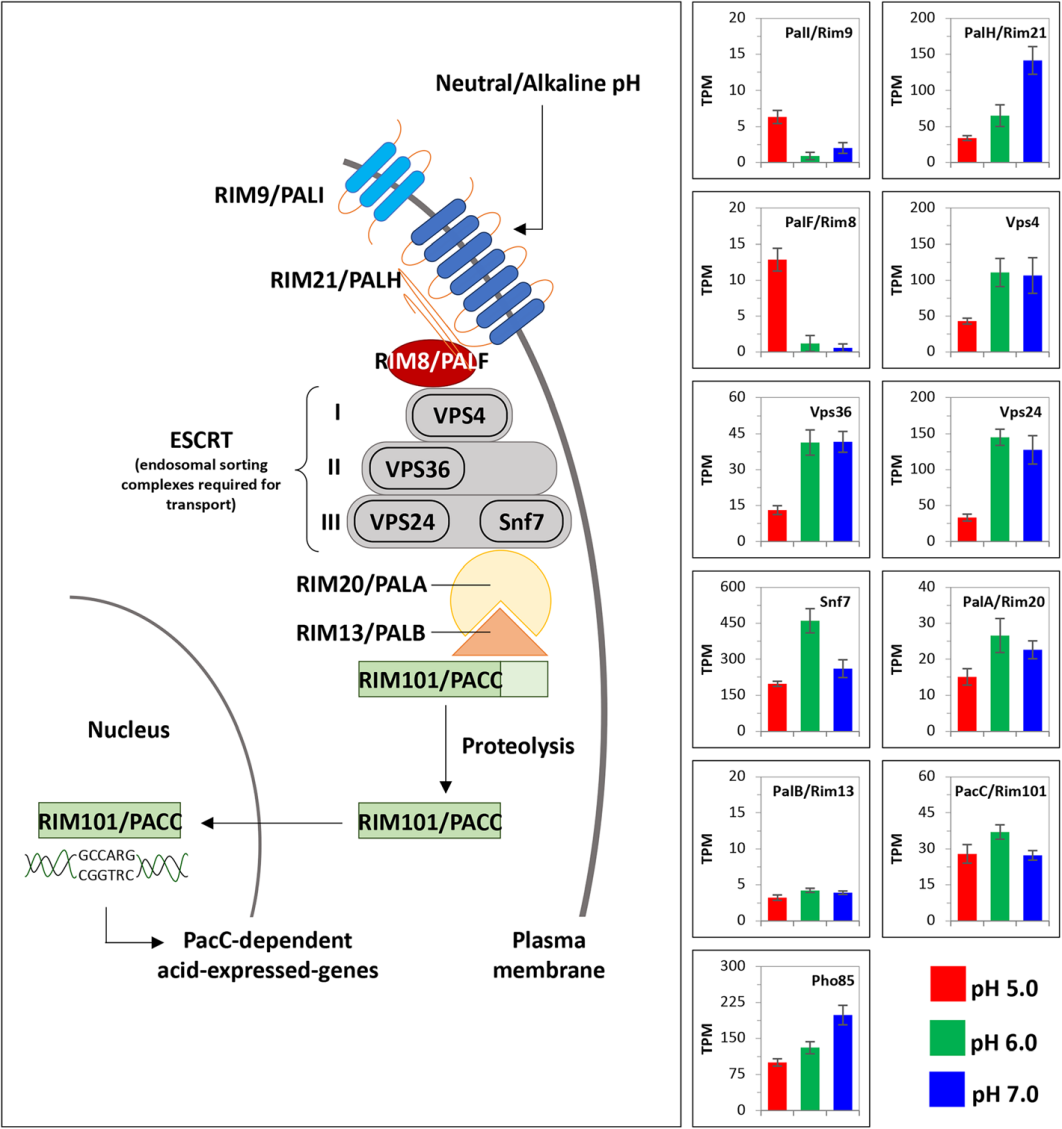
The schematic representation of the canonical Pal/Rim pathway. Differentially expressed *Fusarium* sp. associated with KSBH genes which were identified after comparing the evaluated conditions (pH 5.0, pH 6.0, and pH 7.0) and which are involved in this signaling pathway, are represented in the figure. The left panel shows the expression profiles from each of these nr-unigenes. These expression profiles are represented as transcripts per million (TPM). This figure was modified from [121]. Please note that since transcript levels calculated for each unigenes are different, the Y axis at the left of the bar graphs was scaled to the values of a particular profile

As expected, after surveyed the list of differentially expressed genes, we found many orthologous genes related with the Pal/Rim system and involved in pH signaling. In this sense, Fig. 5 illustrates all these genes in the canonical Pal/Rim pathway [54, 55] and show the expression profiles in *Fusarium* sp. associated with KSBH at each pH condition. PacC, which is a transcriptional activator of alkaline-induced genes and a transcriptional repressor of acid-induced genes [12], was not selected as a differentially expressed unigene; however, considering that is a key player of the PAL/Rim system, we identified the corresponding ortholog in order to analyze its expression profile. The corresponding ortholog (UN05861) shows a similar expression profile in all pH conditions analyzed (Fig. 5 and Additional file 3: Table S8).

PHO85 (UN07212), an additional protein also related to the Pal/Rim pathway was also found in the list of differentially expressed genes showing higher transcript levels at pH 6.0 and 7.0 (Fig. 5 and Additional file 3: Table S8). PHO85 is a cyclin-dependent kinase which plays important roles in a number of cellular responses including carbon source utilization, glycogen metabolism, morphogenesis, cell cycle progression, phosphate metabolism and pH tolerance [56–58].

Additionally, we were interested in identifying genes that showed the greatest fold change value between different pHs, even if they were not the most abundant. In this sense, the nr-unigenes identified as UN01196, UN01849, UN03862, and UN02411 were the genes that most change their expression during the transition from pH 6.0 to 5.0 (upregulated at pH 5.0) with fold-change values (on the log_2_-scale) of 12.36, 12.22, 12.10 and 11.93, respectively. On the other hand, the genes that most change during the transition of pH 5.0 to 6.0 (downregulated at pH 5.0) were: UN01657, UN16350, UN13020 and UN04495 with values of log_2_ fold-change of 12.14, 11.07, 10.85 and 10.54, respectively.

For the nr-unigenes that were most changed during the transition of pH 7.0 to 6.0, the transcriptomic analysis revealed that UN12294, UN07751, and UN06565 are downregulated at pH 7.0 (log_2_ fold-change values of − 10.84, − 9.81 and − 9.08, respectively), while UN11374, UN12042, UN12867, UN02745, and UN10679 are highly upregulated (log_2_ fold-change values of 10.08, 9.83, 9.32, 9.00, and 8.36, respectively; see Additional file 3: Table S8 for more details).

To confirm the expression patterns determined by RNA-sequencing (RNA-seq) analysis, we used qRT-PCR to analyze the expression of eight randomly selected genes. Although the expression values of the eight genes showed slight variations as compared to the corresponding values from the qRT-PCR analyses, the expression profile obtained from the RNA-Seq analysis were highly similar to those obtained from qRT-PCR assays (Additional file 4: Figure S4), indicating that the deep sequencing and qRT-PCR data were consistent.

### Pathogenicity related to environmental alkalization/acidification

**Fig. 6:**
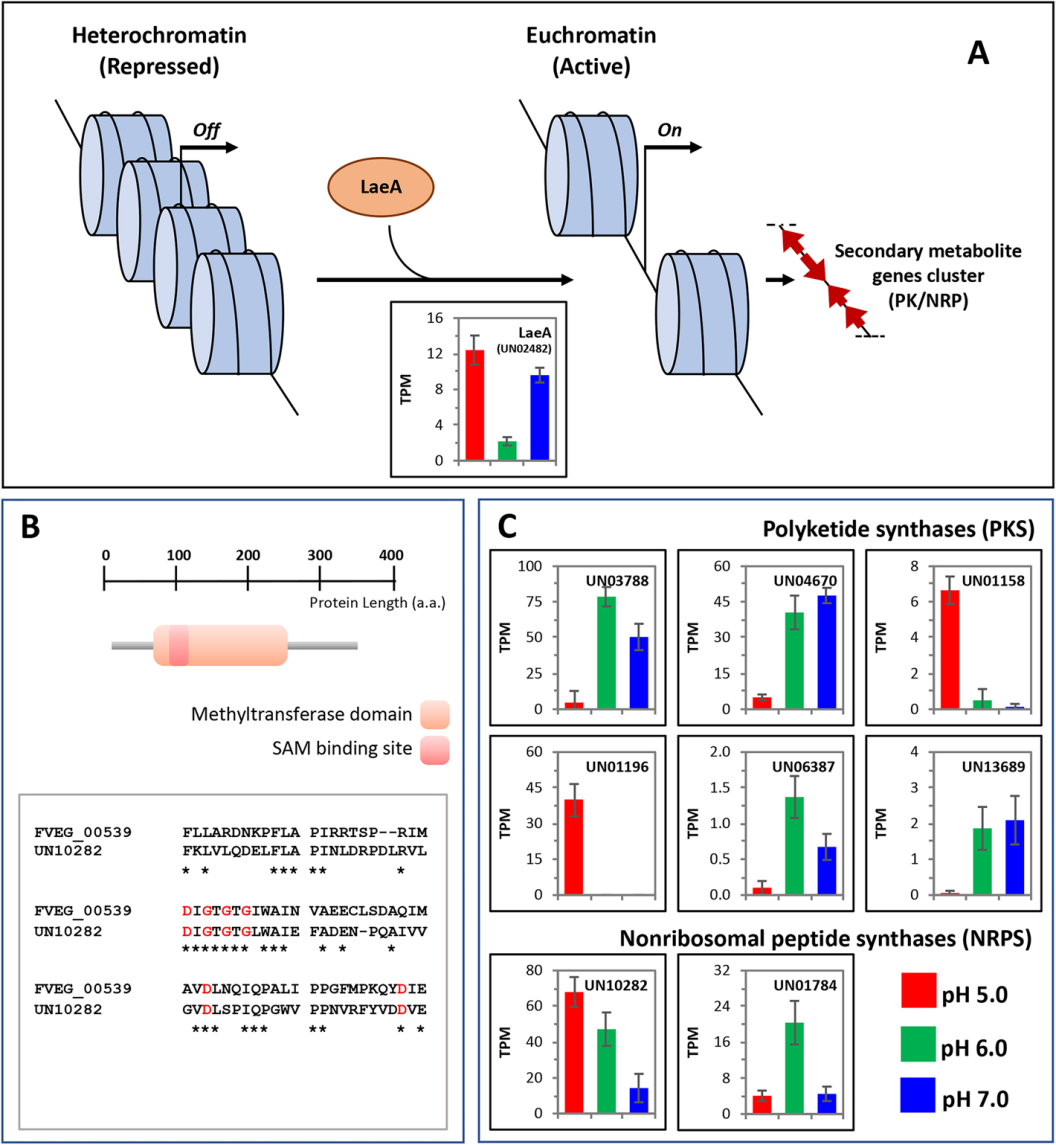
Schematic representation of a putative secondary metabolite genes cluster involved in synthesis of polyketides (PK) or nonribosomal peptides (NRP) which are coded in silenced heterochromatin. This inactive heterochromatin is converted to active euchromatin once LaeA protein interferes with associated methylases or deacetylases, allowing the expression of the genes cluster. **a** The expression profile of a putative LaeA protein of *Fusarium* sp. associated with KSBH at the distinct pH conditions analyzed is shown in the bar graph where transcripts levels are represented in transcripts per million (TPM). **b** Primary structure of *Fusarium* sp. associated with KSBH LaeA protein in which the methyltransferase domain and SAM binding site are represented by orange and red boxes, respectively). Amino acid comparison of LaeA proteins from *F. verticillioides* and *Fusarium* sp. associated with KSBH in which the conserved SAM binding sites are highlighted in red is also presented. Finally, panel (**c**), the expression profiles of each of the *Fusarium* sp. associated with KSBH nr-unigenes identified as homologs to polyketide synthases (PKS1 and FUB1) or nonribosomal peptide synthases (PES1 and NPS6) are shown

It has been suggested that external pH regulates pathogenicity both in a dependent or independent PAL/RIM manner. Because of this, pH-responsive nr-unigenes were compared by BLASTp algorithm against the pathogen-host interaction database (PHI-base) v.3.5 [59]. The PHI-base groups experimentally verified pathogenicity and virulence genes of different pathogens [60] and contain manually curated molecular and biological information, along with the descriptions about mutant phenotypes associated with gene information. Moreover, *F. oxysporum* and *F. graminearum* are comprised in this database [61]. Interestingly, nearly 40% of *Fusarium* sp. associated with KSBH differentially expressed nr-unigenes (1,846 from 4,875), found significant similarity (e-value 10^− 5^) to some of the genes reported in PHI-base (Additional file 3: Table S9), suggesting an important role in pathogenesis (Additional file 4: Figure S5). For instance, transcript levels of LaeA protein (UN02482 and UN02483) increased in all treatments (Fig. 6a). LaeA is a methyltransferase considered a key master regulatory protein of secondary metabolism associated with the epigenetic control.

Both LaeA homologs from *Fusarium* sp. associated with KSBH (UN02482 and UN02483) were 100% identical to each other, and they shared 30% of identity with the protein FVEG_00539. This protein is represented in an *F. verticillioides* microarray which made possible the identification of a putative LaeA homolog of *F. fujikuroi* [62]. Both candidates show a conserved protein methyltransferase s-adenosyl methionine (SAM) binding site, which has been previously identified in LaeA proteins from distinct ascomycetes species [63] (Fig. 6b).

Consistently, homolog genes to polyketide synthases PKS-1 (UN01158, UN03788, and UN04670), and to FUM1 (UN01196, UN06387, and UN13689) were identified as differentially expressed. Both PKS-1 and FUM1, are polyketide synthases (PKS) involved in melanin [64] and fumonisin [65] biosynthesis respectively and have been considered as relevant virulence factors [66, 67]. Regarding nonribosomal peptides, we also found some differentially expressed *Fusarium* sp. associated with KSBH nr-unigenes UN01784 and UN10282 which are homologs to different nonribosomal peptides synthases (NRPS). Expression profiles from PKS and NRPS at the different pH analyzed are shown in Fig. 6c.

The secondary metabolites produced by PKSs or NRPSs, such as the toxins, need to be exported to overcome the intercellular accumulation. Because of this, the fungi have developed efflux pumps powered by ATP-binding cassette transporters (ABC). We found 13 genes differentially expressed that are homologous to ABC-transporters (Additional file 3: Table S9) specifically, the nr-unigenes UN00536, UN00589 and UN03631 were induced at acidic pH.

The host pH modulation has also been considered as a pathogen’s strategy to promote: *i*) better hydrolytic activities, *ii*) permeability increment of the host membranes and/or *iii*) induce the PAL/RIM system described above. In this sense, one of the mechanisms that fungi use to alkalize the medium is through the secretion of ammonia by the glutamate dehydrogenase (GDH2) enzyme. The GDH2 (homolog to UN02168, UN02220, UN02850, and UN02918) is induced at acidic pH. Also, the MEPB transporter has a key role in ammonia secretion, and we found two nr-unigenes (UN11029 and UN12518) that were induced at acidic (pH 5.0 and 6.0, respectively).

Proteases are enzymes that have an important role during infection because these are capable of mediating many cellular functions such as the adhesion of host cells, the initial penetration of the plant cell wall, and colonization and the disease establishment. In this sense, using the PHI database analysis, we obtained 26 nr-unigenes of proteases that have different and particular gene expression among pH conditions (Additional file 3: Table S9).

Finally, an interesting finding was the induction of the putative homolog to the effector GIP2 of *Phytophthora sojae* (UN19266) at neutral pH. The GIP2 is an inhibitor protein that share structural similarity with trypsin class of serine proteases but are nonfunctional. Nevertheless, GIP2 inhibits the soybean endo-β-1,3-glucanase, an enzyme that function as a defense response of the plant. If this finding is biologically functional, it will be the second case that describes a counter defensive weapon used by plant pathogens to suppress a plant defense response [68].

### Identification of *Fusarium* sp. associated with KSBH nr-unigenes with potential relevance to the biosynthetic pathway of fusaric acid

**Fig. 7:**
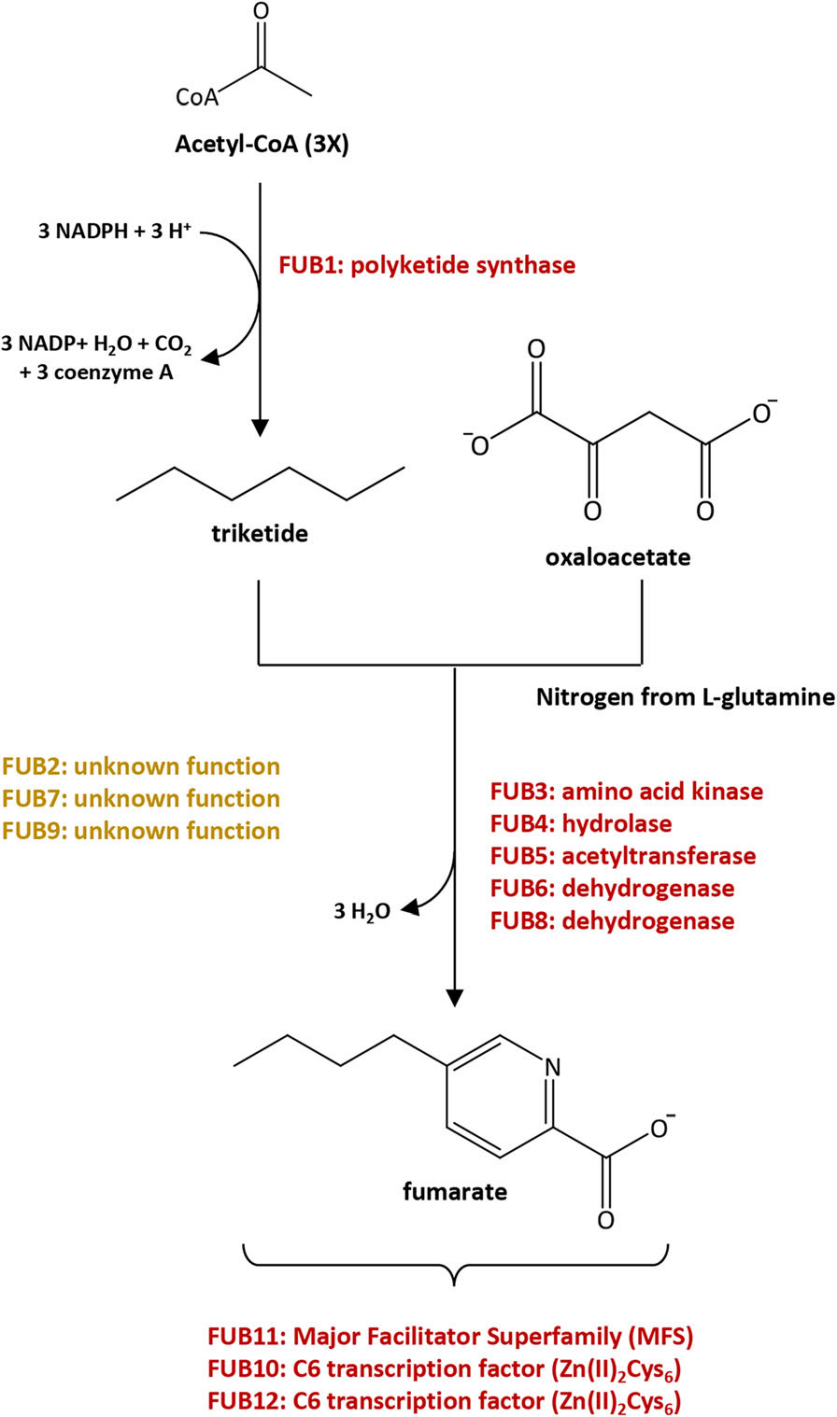
Proposed biosynthetic pathway for fusaric acid biosynthesis in *Fusarium* species. Modified from [98]

The FA biosynthetic pathway in *Fusarium* species was proposed by Brown et al. [69] and comprise a condensation of three acetate units to form a fully reduced 6-carbon polyketide chain which after assimilation of nitrogen from glutamine or oxaloacetate leads to FA formation (Fig. 7). From oxaloacetate to FA, several homologs/orthologs to genes involved in these pathways were identified in the *Fusarium* sp. associated with KSBH transcriptome.

Based on the EC numbers assigned during the annotation process, the seven key enzymes involved in the oxaloacetate biosynthetic pathway were identified in the nr-unigenes: (1) citrate synthase [EC: 2.3.3.1], (2) aconitate hydratase [EC: 4.2.1.3], (3) isocitrate dehydrogenase [EC: 1.1.1.41], (4) succinyl-CoA synthetase [EC: 6.2.1.4], (5) succinate dehydrogenase [EC: 1.3.5.1], (6) fumarate hydratase [EC: 4.2.1.2] and (7) NAD-dependent malate dehydrogenase [EC: 1.1.1.37] (Additional file 4: Figure S6 and Additional file 3: Table S10).

The OrthoMCL-defined protein family in which well characterized FUB1 proteins were grouped comprise a total of 76 orthologous proteins: 12 from *Fusarium* sp. associated with KSBH, 15 from *F. fujikuroi*, 15 from *F. verticillioides*, 12 from *F. oxysporum*, 10 from *F. graminearum* and 12 from *F. solani*, respectively (Additional file 3: Table S11). These data suggest that *Fusarium* species contain a family of PKS which varies slightly in the number of members that compose it. Considering that for *Fusarium* sp. associated with KSBH some of these PKS are represented by partial OFRs, we looked for their complete coding sequences in the genome of *Fusarium* sp. associated with KSBH which has been recently announced [70] and was generated from the same strain used in this study.

**Fig. 8:**
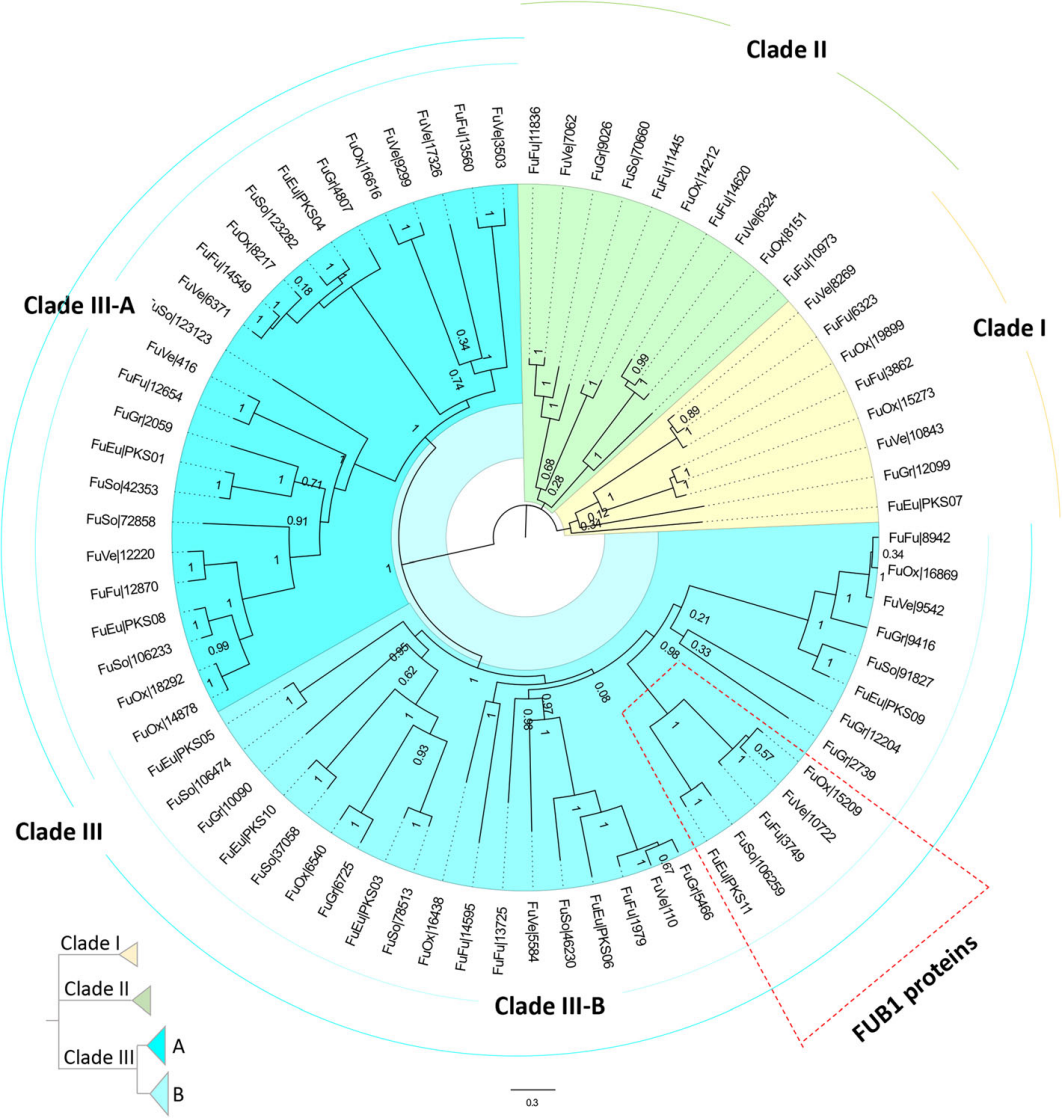
Maximum likelihood phylogenetic trees of polyketide synthases (PKS) protein sequences. Phylogenetic tree of 73 PKS proteins which were identified after OrthoMCL analysis in which six species of *Fusarium* genus were included. The tree was generated using the maximum likelihood method, with the LG substitution model and a BIONJ initial tree. Branch robustness was analyzed by the approximate likelihood-ratio test (aLRT). The members in this phylogenetic tree are grouped into three major clades (identified by roman numbers I to III and highlighted in yellow, green, and cyan, respectively). Of note, clade III can be divided into two sub-clades: Sub-clade A (dark cyan color) and sub-clade B (light cyan color) with the FUB1 clade marked by a red dotted box. Abbreviations: FuFu (*Fusarium fujikuroi*), FuGr (*Fusarium graminearum*), FuOx (*Fusarium oxysporum*). FuSo (*Fusarium solani*), FuVe (*Fusarium verticillioides*) and FuEu (*Fusarium* sp. associated with KSBH). At the bottom-left is a rectangular tree layout in which clades I and II and the sub-clades III-A and III-B are collapsed

**Fig. 9:**
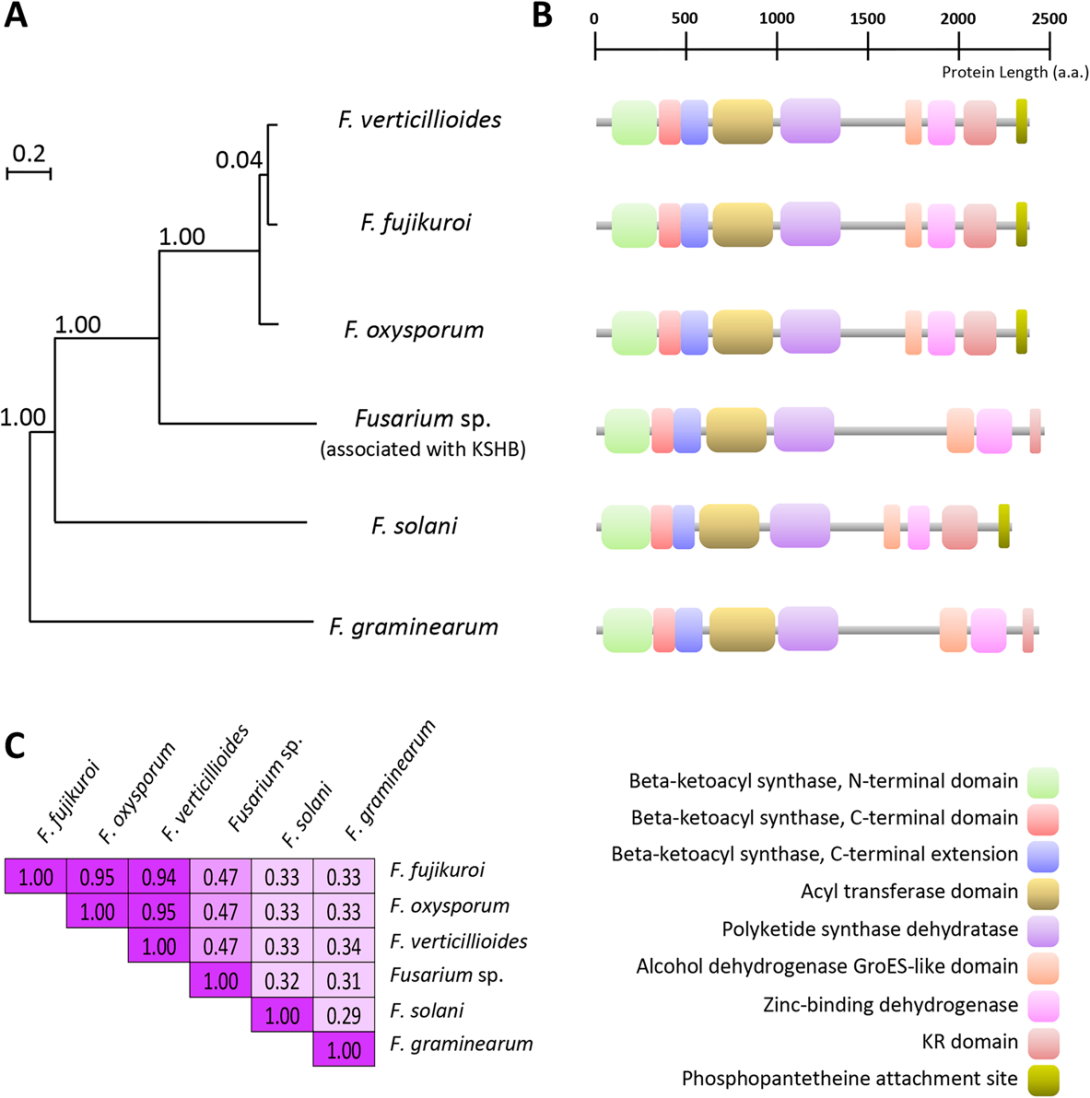
Phylogenetic relationships, primary protein structures and identities percent of FUB1 proteins. **a** Unrooted maximum likelihood phylogenetic tree depicting the relationship between FUB1 proteins from different *Fusarium* species. **b** Primary structures of FUB1 proteins in which the conserved domains (bottom-left panel) are represented by colors boxes. **c** Matrix of the percentage identities between the aligned FUB1 protein sequences

Figure 8 shows the phylogenetic tree constructed by maximum likelihood (ML) for all *Fusarium* PKS proteins which aligned (Additional file 6). Three major clades were recognized on the phylogenetic tree. Clades I and II brought together at least one PKS from most of *Fusarium* species (only *F. solani* was absent on these clades). The third clade (clade III) which included most of the PKS, were spliced in two well-recognized sub-clades (A and B, respectively). Orthologues to FUB1 are contained in clade III-B. These FUB1-like proteins (one for each *Fusarium* species) were independently aligned and represented in a new ML phylogenetic tree (Fig. 9a). The multiple sequence alignments show that FUB1 proteins across *Fusarium* species have identities which ranged from 30 to 95% and they have conserved common motifs which were identified and classified using the Pfam [71] database (Fig. 9b and c).

Regarding the expression profile of the FUB-like *Fusarium* sp. associated with KSBH genes which are orthologous to those reported in other *Fusarium* species, we noticed that none of them were identified as differentially expressed. Interestingly, *fub1*(UN19324), *fub11*(UN12405), and *fub12*(UN05786), show higher transcripts levels at neutral or alkaline pH (pH 6.0 and 7.0) while *fub3*, *fub7,* and *fub9* present greater amounts of transcripts in the acidic treatment (pH 5.0) showing a negative correlation with pH level (Additional file 4: Figure S7). With only one exception (fub1), all FUB-like nr-unigenes from *Fusarium* sp. associated with KSBH show low transcript values (TPM values less than 20). Furthermore, the TPM values for *fub1* are close to zero across all evaluated pH conditions. These results were expected, considering that FA contributes to acidification of the growth medium and the pH was similar in all the treatments when the tissue was harvested to isolate the RNA.

## Discussion

### Identity of the strain used in this study

Based on the phylogenetic analysis, the strain HFEW-16-IV-019 used in this study is grouped with isolates corresponding to *Fusarium* sp. associated with KSBH that were collected in the locations of El Cajon, Fallbrook, Bonsall and Escondido, CA. It is important to mention that in a previous work [70] we named this strain as *F. euwallaceae* because until that time there were not enough genomic data available that allow us to infer the differences between the *Fusarium* strains associated with KSBH or PSHB [21].

### Effect of pH on conidiation and growth of *Fusarium* sp. associated with KSHB

Acidic/alkaline requirement for fungi growth is quite broad, ranging from pH 3.0 to more than pH 8.0, with the optimum around pH 5.0 if nutritional requirements are satisfied [72]. Some *Fusarium* species (e.g. *F. oxysporum*), have shown the ability to grow in acid conditions (pH 2.0) with an optimal value of pH 6.0 for growth and sporulation [73]. In contrast, our results showed that a highly acid medium (pH 3.0 or less) completely inhibited the growth of *Fusarium* sp. associated with KSHB*.*

### Differential massive expression analysis of pH-responsive genes in *Fusarium* sp. associated with KSBH

Hierarchical clustering and heatmap analyses show similar expression patterns with subtle changes between the pH 6.0 or pH 7.0 treatments. Interestingly, both treatments show significant differences when they are compared with pH 5.0 (Fig. 4 and Additional file 3: Table S7). These data suggest that molecular responses of *Fusarium* sp. associated with KSBH are similar when the fungus is growing at pH 7.0 or 6.0, while significant differences can be observed when the fungus is growing at pH 5.0.

The fungi have developed a signal transduction pathway in order to sense external pH designated as RIM or PAL for yeasts or filamentous fungi, respectively. We found many orthologous genes related with the Pal/Rim system and involved in pH signaling during the differential massive analysis in *Fusarium* sp. associated with KSBH. The activation of the PAL/Rim system requires firstly the perception of the external pH by a molecular complex located in the plasma membrane that includes PalH/Rim21 (UN01086) a 7-transmembrane domain receptor which senses alkaline pH [74] and facilitate ubiquitination of the arrestin-like protein PalF/Rim8 (UN03271) [75]. These proteins, as well as the chaperone PalI/Rim9 protein (UN01473), are a component of the membrane sensing complex [76]. PalF/Rim8 binding to the VPS23 protein (ESCRT-I) induces the assembly of the ESCRT-II and -III complexes as a scaffolding platform for Pal/Rim pathway activation [77, 78]. On this platform, the Pal/Rim proteolysis complex is assembled by PalA/Rim20 (UN07027), and the PalB/Rim13 protease (UN01037) [79]. This proteolysis complex catalyzes cleavage and activation of its target, the PacC/Rim101 transcription factor (UN05739) which acts directly as transcriptional activator of “alkaline” genes [80]. According to the expression profile of the orthologous genes identified in *Fusarium* sp. associated with KSBH (Fig. 5 and Additional file 3: Table S8), PALH/RIM21 receptor is upregulated in neutral/alkaline pH just like other proteins related to the endosomal sorting complexes required for transport (ESCRT I, II, and III, respectively). However, proteins from the proteolysis complex show different expression profile. On the one hand, PalA/Rim20 is also upregulated at neutral/alkaline pH, while PalB/Rim13 should be considered as ubiquitous because even when its expression profile is similar to others upregulated genes, the ortholog to PalB/Rim13 of *Fusarium* sp. associated with KSBH did not show significant values after DEseq normalization (Fig. 5 and Additional file 3: Table S8). This was expected since the pH of all the samples had decreased to an acidic level at the time of sample collection. PacC also shows lowest transcript levels in comparison with some other genes of the PAL/Rim system (e.g. PalH, Vsp4, Vsp24 and Snf7). This data suggest that the basal transcript levels detected for PacC could be related with some other functions performed in addition of perception and signaling to the alkaline conditions. For example, it has been reported that PacC null mutant is comprised in growth and conidiation in *Aspergillus* [12]. The fact that *Fusarium* sp. associated with KSBH, grew at initially acidic conditions (media pH 5.0) and even after 2 weeks the pH decreased more, suggests two alternatives: first an active Pal/Rim route, and second, an alternative regulation and/or adaptation mechanism of the fungus to grow at high concentration of hydrogen ions but with some restrictions since the fungus was not able to grow at pH 3.0. Together, these data show that the PAL/RIM pathway is present in *Fusarium* sp. associated with KSBH.

Also, we identified genes that showed the greatest fold change value between different pHs.

The first group were the genes that most change their expression during the transition from pH 6.0 to 5.0 (UN01196, UN01849, UN03862, and UN02411). UN01196 gene encodes for a polyketide synthase protein which was homologous to FUB1, the importance of this protein and its role in pathogenicity is discussed below. UN01849 is a homolog of the vacuolar segregation 7 protein (Vac7), which in yeast functions as a Fab1 activator and together forms part of the Phosphatidylinositol 3,5-bisphosphate [PI(3,5)P2] regulation system. The PI(3,5)P2 system is critical for acidification of the vacuole since experiments with *fab1*Δ and *vac7*Δ mutants showed lower acidification of the vacuole compared to the wild-type [81]. UN03862 is a hypothetical protein and has a multidrug and toxic compound extrusion (MATE) domain. The MATE transporters are implicated in the multidrug resistance through the exclusion of xenobiotics, and toxic metabolites from cells only have been studied in *Saccharomyces cerevisiae* and confers resistance to the methionine analog ethionine [82]. UN02411 is predicted to be similar to a serine carboxypeptidase F from *Fusarium solani* F33, and the enzyme can release arginine and lysine from the carboxy terminus of peptides [83].

UN01657, UN16350, UN13020 and UN04495 were the genes that most change during the transition of pH 5.0 to 6.0 (downregulated at pH 5.0). UN01657 encodes a carnitine O-acetyltransferase, UN016350 a 2,3- dihydroxybenzoate decarboxylase, UN13020 a succinate-fumarate transporter and UN04495 an aldo-keto reductases. Both up- and down-regulated genes at pH 5.0 are included in the Additional file 3: Table S8.

The nr-unigenes UN12294, UN07751, and UN06565 were downregulated at pH 7.0 (during the transition of pH 7.0 to 6.0), while UN11374, UN12042, UN12867, UN02745, and UN10679 were highly upregulated.UN12294 encodes a 2-dehydropantoate 2-reductase, this enzyme participates in pantothenate (Vitamin B_5_) biosynthesis pathway. UN07751 is recognized as an amine oxidase such as a polyamine oxidase and UN06565 encodes for an unknown protein that present two domains assigned as fungal Zn binuclear cluster domain containing protein and viral late glycoprotein. With respect with the nr-unigens that are highly upregulated both UN11374 and UN10679 encode a type of permease, for example, the first one encodes an α-glucoside permease and the second one a related to neutral amino acid permease. UN12867 is an uncharacterized protein, UN02745 codes for a low-affinity potassium transporter protein, and UN12042 a coatomer subunit delta. Interestingly, all these genes encode proteins that are related to transport through the membranes and suggest their role during an efficient metabolites mobilization as well during the adjustment of pH condition of the medium. This observation is based in part on the existence of a readjustment of the initial conditions of the medium, which changed to acidic conditions after 2 weeks of growth of *Fusarium* sp. associated with KSBH. Finally, it is worth mentioning that the global transcriptomic analysis at each condition showed clear differences independent of the high concentration of hydrogen ions prevailing at the end of all the experiments. Interestingly, this suggests that we could use the transcriptional expression profile to know the immediate past ecologic niche of the fungus.

### Pathogenicity related to environmental alkalization/acidification

In order to identify genes related with pathogenicity in *Fusarium* sp. associated with KSBH, pH-responsive nr-unigenes were compared by BLASTp algorithm against the pathogen-host interaction database (PHI-base) v.3.5 [59]. In this sense various genes were recognized such as LaeA gene (Fig. 6). LaeA is a methyltransferase considered a key master regulatory protein of secondary metabolism and involved in an initial process that converts heterochromatin to euchromatin, perhaps by interfering with methylases or deacetylases associated with heterochromatin. Commonly, secondary metabolites are codified by gene clusters which are maintained as silenced heterochromatin, but, under certain stimulus, the chromatin is reprogrammed to result in increased transcription of these secondary metabolite genes [84]. It has been proved that deletion of LaeA gene results in widespread reduction of most secondary metabolites, mainly polyketides and nonribosomal peptides including metabolites with toxic properties [85]. In addition, in *F. graminearum*, it was revealed that the FgLaeA is a positive regulator of mycotoxin production such as trichothecene and zearalenone [86].

Likewise, homolog genes to PKS-1 and to FUM1 were differentially expressed. Both PKS-1 and FUM1, are polyketide synthases (PKS) involved in melanin [64] and fumonisin [65] biosynthesis respectively and have been considered as relevant virulence factors [66, 67]. It has been reported that disruption of pks1 gene reduced virulence in *Cochliobolus heterostrophus - Zea mays* interaction assays [87] while some fumonisins, mainly, fumonisin B1, is recognized as a virulence factor since inhibits three different plant targets: *i*) the sphinganine N-acyl transferases, disrupting sphingolipid metabolism (for review [88]), *ii*) the maize plasma membrane H + -ATPase [89] and *iii*) certain maize β-1,3-glucanase isoforms [90].

Regarding nonribosomal peptides, we identified some differentially expressed such as PES1 (UN10282), that is an NRP synthetase which confers protection against oxidative stress [91] and is essential for Fumigaclavine C production in *Aspergillus fumigatus* [92]. Also, a homolog to NPS6 (UN01784) was also found as a remarkable NRP related with the pathogenesis since is a conserved virulence factor of plant pathogenic ascomycetes involved in siderophore-mediated iron metabolism [93]. Also, the NRPS are responsible for beauvericin synthesis, a *Fusarium* mycotoxin that induces cell death in tomato protoplasts [94].

Moreover, we identified 13 nr-unigenes homologous to ABC-transporters. Many of these transporters are responsible also for exporting the host-derived antimicrobial compounds, so the transporters, undoubtedly, have a crucial role in the pathogenesis [95]. Interestingly, it seems that UN00536 is homolog to the ABC transporter ABC4 from *Magnaporthe grisea*. The abc4 mutant did not form functional appressoria and is nonpathogenic [96]. Also, we found two *Fusarium* sp. associated with KSBH nr-unigenes (UN00589, UN03631) that are homologs to ABC1 transporter of *F. graminearum*. It has been reported that FgABC1 and FgABC3 deletion mutants were impeded in virulence on wheat, barley, and maize [96].

We also recognized, 26 nr-unigenes of proteases that have differential expression among the treatments. Is already known de importance of this kind of enzymes during pathogenesis and we found that the nr-unigenes UN05400 and UN06203 have homology with BcNma, a protease of *B. cinerea* involved in apoptosis and viability, interestingly, the mutant has slightly reduced virulence [68]. Also, we identified a protease that was induced at neutral pH, and it is the homolog of a vacuole-localized protease named SPM1 (UN12426) that is a protease required for the infection of the rice blast fungus *Magnaporthe oryzae* [97].

### Identification of *Fusarium* sp. associated with KSBH nr-unigenes with potential relevance to the biosynthetic pathway of fusaric acid

Fusaric acid (FA) is a polyketide-derived secondary metabolite which is produced by multiple *Fusarium* species (revised by [69, 98, 99]). FA (or 5-butylpicolinic acid), acidifies plant surfaces [7], elicit plant defense responses [100], induces programmed cell death [9] and it has been strongly associated with plant wilt symptoms [100]. Moreover, it has been reported that in several species such as *F. verticillioides*, *F. fujikuroi* and also different strains of *F. oxysporum*, genes related to FA production and fumonisins are clustered, and both clusters (FUB and FUM) are uniform in gene organization [98, 101].

Despite small variants in the genomic regions from distinct species, a total of 12 genes are present in FUB cluster. Even when FUB genes are coexpressed during specific growth conditions (e.g. GYAM medium at pH 3.0, [69]), specific roles from some FUB genes during fusaric acid biosynthesis [69] and some of the biochemical mechanism by which fusaric acid is biosynthesized from polyketide precursors remains unknown or are speculative [69, 98].

Regarding the FA biosynthetic pathway, the *Fusarium* sp. associated with KSBH orthologous proteins were identified by searching nr-unigenes grouped with FUB proteins that have been previously characterized in others *Fusarium* species (Additional file 3: Table S11). Orthologues to FUB1, FUB3, FUB7, FUB9, FUB11, and FUB12 were identified, while the remaining FUB orthologous proteins (FUB2, FUB4–6, FUB8, and FUB10) were absent in the *Fusarium* sp. associated with KSBH transcriptome. Orthologues to the same FUB proteins were also absent in *F. solani* and *F. graminearum* species (Additional file 3: Table S11). These data suggest that maybe in some *Fusarium* species, not all FUB genes are essential to produce this toxin. This is consistent with the observations about the FUB genes present in *F. solani*, a closely related species to *Fusarium* sp. associated with KSBH in which the ability to produce fusaric acid has been proved experimentally [102]. Also, even when the FUB cluster consists of at least 12 genes, analysis of deletion mutants has been shown that only nine FUB genes are required for fusaric acid production and only the deletion of some of them can partially or completely arrest the synthesis of this metabolite [98]. For instance, in *F. fujikuroi*, *fub3* and *fub5* deletion reduced fusaric acid production by 20% and 40%, respectively [103]. Meanwhile, deletion of *fub1* (which encodes a PKS) completely arrested the fusaric acid production in both *F. verticillioides* and *F. fujikuroi* [69, 103]. Recently, it has been reported that *fub1* in *F. oxysporum* is positively controlled by LaeA and PacC through the modulation of chromatin accessibility at the *fub1* locus [104].

## Conclusions

The ability to acidify the external environment is shared by some pathogenic fungi and might contribute to generating an appropriate environment to produce some virulence factors which contribute to damaging the host tissues and promote the development of the disease. For instance, plant-necrotizing fungi such as *Sclerotiorum clerotinia* and *Botrytis sp*., secrete significant amounts of oxalic acid [14], while *Penicillium sp*. and *Aspergillus sp*. secrete mainly gluconic and citric acids [105, 106]. The produced acids not only acidify the tissues but can also lower the activity of reactive oxygen species produced by the host [107]. The fusaric acid produced by some *Fusarium* species acidifies plant surfaces and activates the membrane H+ -ATPase, a pH-regulated process that leads to the expression of proteases and subsequent tissue invasion [7]. In contrast, other fungi can alkalize the environment by the active secretion of ammonia. This capability has been recognized in *Colletotrichum* species, *Fusarium oxsyporum*, and *Alternaria alternata* [10]. This work shows that *Fusarium* sp. associated with KSBH has the machinery to adapt to acidic environment. It will be necessary in the future to determine if this is useful for its virulence and to establish disease. Besides, the behavior of the fungus must be evaluated at alkaline conditions. Finally*, Fusarium* sp. associated with KSBH has the ability to induce transcription of genes related to virulence such as proteases, PKs, ABC transporters at both alkaline pH and acidic pH. The plasticity that is observed in this fungus to adapt to such broad pH conditions suggests that it can be a difficult pathogen to treat.

## Methods

### Biological material

The ‘Secretaría de Agricultura, Ganadería, Desarrollo Rural, Pesca y Alimentación (SAGARPA)’ through ‘Servicio Nacional de Sanidad, Inocuidad y Calidad Agroalimentaria (SENASICA)’ and the ‘Dirección General de Sanidad Vegetal (DGSV)’ provided the biological material and the corresponding permission to work with the fungus in the ‘Centro Nacional de Referencia Fitosanitaria (CNRF)’ facilities under strict biosecurity conditions. The fungus *Fusarium* sp. associated with KSBH was isolated from the mycangia of beetles collected from internal galleries found in elm (*Ulmus* sp.) trees identified by Mexican phytosanitary authorities in urban areas of Tijuana municipality showing typical symptoms caused by the polyphagous shot hole borer and by their associated fungi [18, 20, 49, 108]. The beetle was taxonomically identified, and the species of the fungus was confirmed by searching for and aligning four common markers previously used in phylogenetic analysis to distinguish between different strains of *Fusarium* sp. associated with KSBH which were isolated from various sources and locations. For additional information see [20] and the corresponding section described above (Phylogenetic relationship with additional *Fusarium* sp. associated with KSBH strains). The four loci were: LSU (nuclear ribosomal large subunit), EF-1α (translation elongation factor 1a), RPB1 and RPB2 (subunits 1 and 2 of the DNA-directed RNA polymerase II).

### Growth conditions

Potato Dextrose Agar (PDA) medium was used for maintenance of stock cultures. For inoculum preparation, the fungus was initially grown at 28 °C on a PDA plate for 7 days. Then, a 0.7 cm^2^ plug from the outer zone of the colony was punched with a sterile well cutter and transferred to 100 mL Potato Dextrose Broth (PDB) medium contained in a 250 mL Erlenmeyer flask. Before autoclaving, the pH of the medium was adjusted to specific levels using either 10 N HCl or 10 N NaOH. The fungus was grown at 28 ± 2 °C on a rotary shaker at 200 rpm, and three replicate sets were grown in each case. After 2 weeks (14 days), the culture broth was filtered through a filter paper (Whatman No.1), and the collected fungus was pulverized cryogenically using a mortar and pestle.

### RNA isolation, library preparation, and sequencing

Total RNA was isolated from 200 mg homogenized tissue according to the manufacturer’s protocol and using a Norgen RNA Purification Kit (Norgen Biotek Corporation). The RNA integrity was evaluated by chip-based capillary gel electrophoresis using a Bioanalyzer system (Agilent Technologies). The RNA concentration was determined by absorbance at 260 nm using a NanoDrop 2000 UV-Vis spectrophotometer (Thermo Fisher Scientific). A total amount of 500 ng of RNA was used as input material for each RNA sample preparation. Nine libraries (three independent biological replicates per analyzed conditions: pH 5.0, 6.0 and 7.0, respectively) were generated using the TruSeq RNA Sample Preparation Kit (Illumina, Cat. N°RS–122–2002) following manufacturer’s recommendations and index codes were added to identify each sample independently. The libraries were sequenced on the NextSeq 500 platform (Illumina) using the 2 × 150 bp paired-end sequencing protocol. The sequence images were transformed to bcl files with Illumina NextSeq control software (v1.4) and Real-Time Analysis software (v2), then bcl files generated were demultiplexed to fastq files with bcl2fastq (v2.0) conversion software. Files containing sequence reads and quality scores were deposited in the Short Read Archive (SRA) of the National Center for Biotechnology Information (NCBI). Accession number SRP156234.

### Quality control of sequenced reads and de novo transcriptome assembly

Before assembly, raw data in fastq format were processed through a Python-based script available at GitHub repository (https://github.com/Czh3/NGSTools/blob/master/qualityControl.py). The stringency parameters employed to select High Quality (HQ) paired-end reads were: -q 20 (Minimum quality score to keep), −p 90 (Minimum percent of bases that must have [−q] quality) and -a 30 (the minimum average quality in the paired-end reads selected). SeqPrep v1.1 (https://github.com/jstjohn/SeqPrep) was used to remove adapters and merge HQ paired-end reads with overlap into a single longer reads. Adapter sequences were trimmed by searching for a ≥ 10 nt overlap between the end of each read and the adapter sequence allowing up to 2% mismatched bases of superior quality (≥ 20) and requiring a minimum of 87% matching bases in the overlapping region. Reads were discarded if they were shorter than 75 bp and orphan reads were also removed to keep pairs only. Forward and reverse reads were merged into single reads if they overlapped for at least 25 nt allowing up to 2% mismatching bases of superior quality and requiring a minimum of 90% matching bases in the overlapping region. Transcriptome de novo assembly was performed including adapters-free HQ paired-end reads and those longer sequences resulting from merging reads (R1 and R2) through their overlapping regions. Trinity assembler [109] with default parameters was used for this purpose in which all data set generated (libraries from pH 5.0, 6.0, and 7.0, and their corresponding replicates) were combined. Finally, the contigs resulting from the assembly process (also commonly named as unique transcripts or unigenes) were masked using the SeqClean software to eliminate sequence regions that could cause problems during the annotation process. Targets for masking include poly A/T tails and end rich with Ns (undetermined bases) and/or low complexity sequences. A unigenes set from *Fusarium* sp. associated with KSBH was generated considering only resulting contigs with a minimum length of 200 bp.

### Identification of protein coding regions, and annotation

To generate an accurate and non-redundant data set representative from the *Fusarium* sp. associated with KSBH transcriptome, erroneous frame-shifts identified within the coding region were corrected based on the alignment of each assembled unigene against its homologs (at least three) identified by BLAST searches. AlignWise [26], a pipeline which drives several programs such as BLAST ([27]; for similarity searches), Muscle ([110] to lead the alignments) and GeneWise ([111] to identify ORF and correct frame-shifts), was used for such purposes. Once the ORF nucleotide sequences were identified and corrected for potential frame shifts, the BLASTClust program [112] was used to create a non-redundant set of sequences. Assembled sequences 95% identical over an area covering at least 90% the length of another unigene, were removed to avoid redundancies.

BLAST searches were performed to identify homologs to the proteins translated from coding regions identified in the nr-unigenes for each set of reference proteins, those. BLASTp algorithm [27] was used for such purposes. An E-value threshold of 10^− 5^ was considered in all comparisons. The latest version for all reference proteins was downloaded from JGI/MycoCosm portal (http://genome.jgi.doe.gov/programs/fungi/index.jsf). The selected fungi species were mentioned in the Results section above. Additionally, in order to classify the nr-unigenes based on a functional annotation, InterPro (IPR) domains, the Gene Ontology (GO) terms, and the EuKaryotic Orthologous Groups (KOG) derived from Kyoto Encyclopedia of Genes and Genomes (KEGG), were assigned based on the information available for the homologous proteins which were identified in BLAST searches using the SBH (single-directional best hit) method.

### Ortholog groups identification

The complete proteomes encoded by the genomes of *S. cerevisiae*, *F. fujikuroi*, *F. oxysporum*, *F. verticillioides*, *F. graminearum*, and *F. solani* as well as the proteins produced after translated the ORFs identified in the *Fusarium* sp. associated with KSBH nr-unigenes, were globally compared. The complete data set represents 111,688 protein-coding sequences (5,982 from *S. cerevisiae*, 14,813 from *F. fujikuroi*, 13,322 from *F. graminearum*, 20925 from *F. oxysporum*, 15,707 from *F. solani*, 15,869 from *F. verticillioides*, and 25,070 from *Fusarium* sp. associated with KSBH). Except for *Fusarium* sp. associated with KSBH, only proteins translated from representative gene models (without alternative isoforms) were included. An all-against-all comparison was performed using BLASTp (threshold E-value of ≤10^− 10^) followed by clustering with the v2.0.9 of OrthoMCL program [113]. Default parameters, including an inflation value of 1.5, were used to define orthologous cluster structure.

### Phylogenetic analyses

Phylogenetic analyses of PKS proteins were performed in a maximum-likelihood (ML) framework using SeaView v2.4 software [114], which drives the Muscle [110] (for alignment) and PhyML [115] (for phylogenetic analysis) programs. The PhyML option was used under LG (Le and Gascuel) model [116]. Equilibrium frequencies, topologies, and branch lengths were optimized, the starting tree was determined using BioNJ, and both nearest-neighbor interchange (NNI) and subtree pruning and regrafting (SPR) algorithms for tree searching were used. Branch robustness was analyzed by approximate likelihood-ratio test (aLRT) [117].

### Identification of differentially expressed genes

The pH-responsive genes were identified by comparing global expression profiles obtained from each transcriptomic data set of *Fusarium* sp. associated with KSBH generated for the three analyzed conditions (pH 5.0, 6.0, and 7.0). First, the high-quality reads from each sample were mapped to the reference transcripts (unigenes) using RSEM software [50]. RSEM is a software package that use short reads mappers such as Bowtie2 [118] to independently assign the reads generated from each condition (distinct pH levels) to each of the unigenes resulting from the assembly process. The number of reads assigned to a specific unigene represents the abundance of mRNA produced by a particular gene in a given sample. The Expectation-maximization algorithm implemented in RSEM returns an expression profile matrix where the transcript abundance is represented by the expected reads counts (a non-normalized value) and their normalized values, TPM (transcripts per million) and FPKM (fragments per Kilobases of contigs/unigenes for per million mapped reads). Expected reads counts are then imported by tximport package [119] to be then used as input for DESeq, an R/Bioconductor package [51] which normalize the samples comparing them by pairs and using the relative log expression method to adjust the ratio values of the gene expression levels to baseline, giving the hypothesis that most genes are not differentially expressed. After comparisons of paired samples (pH 5.0/pH 6.0; pH 5.0/pH 7.0 and pH 7.0/pH 6.0), an adjusted *P*-value of < 0.05 was considered as threshold to identify differentially expressed unigenes.

### Gene expression validation using real-time PCR

According to the manufacturer’s instructions, cDNA templates for PCR amplification were prepared from all samples by using reverse specific primers and SuperScript III reverse transcriptase (Invitrogen). Each quantitative PCR reaction contained cDNA template from ~ 1 μg total RNA, 1× SYBR Green® PCR Master Mix (Life Technologies) and 500 nM forward and reverse primers (Additional file 3: Table S12). Real-time PCR was performed on an STRATAGEN MX3005P (Agilent Technologies) detection system under the following thermal cycling conditions: 10 min at 95 °C followed by a total of 40 cycles of 30 s at 95 °C, 30 s at 55 °C and 1 min at 72 °C. For qRT-PCR, relative transcript abundance was calculated and normalized with respect to Pol II (DNA-directed RNA polymerase II core subunit RPB2; UN04406) a commonly used housekeeping gene which permits minimize variation in cDNA template levels. All calculations and analyses were performed using MxPro PCR software (Agilent Technologies) and the 2^−ΔΔCt^ method [120]. Amplification efficiency (85.49 to 101.56%) for the primer sets was determined by amplification of cDNA dilution series (1:5). Specificity of the RT-PCR products was followed by a melting curve analysis with continuous fluorescence data acquisition during the 55–95 °C melt.

## Additional files

Additional file 1: **Table S1.** Summary of sequencing data generated from *Fusarium* sp. associated with KSHB *Euwallacea* sp. near *fornicatus*. **Table S2.** Annotation of Fusarium sp. associated with KSHB *Euwallacea* sp. near *fornicatus* no-redundant unigenes. (XLSX 25088 kb)

Additional file 2: The nucleotide sequence of the non-redundant unigenes from *Fusarium* sp. (ZIP 20501 kb)

Additional file 3: **Table S3.** Functional categorization of the *Fusarium* sp. associated with KSHB *Euwallacea* sp. near *fornicatus* non-redundant unigenes **Table S4.** Metabolomic pathways network. **Table S5.** OrthoMCL-defined protein families shared between some Ascomycota species. **Table S6.** Expression profile matrix. **Table S7.** pH-responsive genes identified in *Fusarium* sp. associated with KSHB *Euwallacea* sp. near *fornicatus*. **Table S8.** pH-responsive genes from *Fusarium* sp. *associated* with KSHB *Euwallacea* sp. near *fornicatus* involved in specific matabolic pathways. **Table S9.** pH-responsive genes from *Fusarium* sp. associated with KSHB *Euwallacea* sp. near *fornicatus* with significant similarity (e-value 10^-05^) to those genes/proteins previosly reported in PHI-base (pathogen-host interaction database). **Tabla S10.** Non-redundnat unigenes from *Fusarium* sp. associated with KSHB *Euwallacea* sp. near *fornicatus* involved in the tricarboxylic acid cycle (oxaloacetate biosynthetic pathway). **Table S11.** Genes involved in fusaric acid (FA) biosynthesis. **Table S12.** Primers used in RT-qPCR assays. (XLSX 14848 kb)

Additional file 4: **Figure S1.** Metabolic network (and their corresponding pathways) represented in the nr-unigene set from *Fusarium* sp. associated with KSBH *Euwallacea* sp. near *fornicatus*. **Figure S2.** Comparison of *Fusarium* species proteomes. **Figure S3.** Maximum likelihood phylogenetic tree based on the concatenated sequences of four-locus analyzed (RB1, RB2, EF-1a, LSU). **Figure S4.** Results of gene expression validated by quantitative real-time PCR analysis. **Figure S5.** GO treemap for the differentially expressed genes and related to pathogen-host interaction. **Figure S6.** Oxaloacetate biosynthesis pathway reconstructed based on the de novo assembly and annotation of the *Fusarium* sp. associated with KSBH *Euwallacea* sp. near fornicatus transcriptome. **Figure S7.** Proposed biosynthetic pathway for fusaric acid biosynthesis in species of *Fusarium* sp. associated with KSBH *Euwallacea* sp. near *fornicatus* and the expression profile of genes involved in the pathway [122, 123]. (PPTX 5498 kb)

Additional file 5: Concatenated multi-locus sequence alignment used in Maximum Likelihood phylogenetic tree. (FASTA 184 kb)

Additional file 6: Alignment of the full-length sequences of the polyketide synthases (PKSs) from distinct *Fusarium* species. (FASTA 1114 kb)

## Abbreviations

ABC: ATP-binding cassette transporters
AFC: Ambrosia Fusarium Clade
aLRT: Approximate Likelihood-Ratio Test
BLAST: Basic Local Alignment Search Tool
EC: Enzyme Commission (Number)
FPKM: Fragments Per Kilobases of Contigs/Inigenes for Per Million Mapped Reads
FSSC: *F. solani* Species Complex
IPR: InterPro (Domains)
ITS: Internal Transcribed Spacer (Region)
KSHB: Kuroshio Shot Hole Borer
MCL: Markov CLuster (Algorithm)
NNI: Nearest Neighbour Interchange
NRPS: Non-Ribosomal Peptides Synthases
ORFs: Open Reading Frames
PDA: Potato Dextrose Agar
PDB: Potato Dextrose Broth
PKS: Polyketide Synthases
SAM: S-adenosyl methionine (binding site)
SBH: Single-Directional Best Hit (method)
SPR: Subtree pruning and regrafting (algorthims)
SRA: Short Read Archive
TPM: Transcripts Per Million
