# Supplementary material for: Environmental pH modulates transcriptomic responses in the fungus Fusarium sp. associated with KSHB Euwallacea sp. near fornicatus
Source: BMC Genomics. 2018 Oct 1;19:721. doi: 10.1186/s12864-018-5083-1 (PMC6167834; doi:10.1186/s12864-018-5083-1)
Supplement: Supplementary file 4 — Figure S1. Metabolic network (and their corresponding pathways) represented in the nr-unigene set from Fusarium sp. associated with KSBH Euwallacea sp. near fornicatus. Figure S2. Comparison of Fusarium species proteomes. Figure S3. Maximum likelihood phylogenetic tree based on the concatenated sequences of four-locus analyzed (RB1, RB2, EF-1a, LSU). Figure S4. Results of gene expression validated by quantitative real-time PCR analysis. Figure S5. GO treemap for the differentially expressed genes and related to pathogen-host interaction. Figure S6. Oxaloacetate biosynthesis pathway reconstructed based on the de novo assembly and annotation of the Fusarium sp. associated with KSBH Euwallacea sp. near fornicatus transcriptome. Figure S7. Proposed biosynthetic pathway for fusaric acid biosynthesis in species of Fusarium sp. associated with KSBH Euwallacea sp. near fornicatus and the expression profile of genes involved in the pathway [122, 123]. (PPTX 5498 kb) [file 12864_2018_5083_MOESM4_ESM.pptx]

## Slide 1
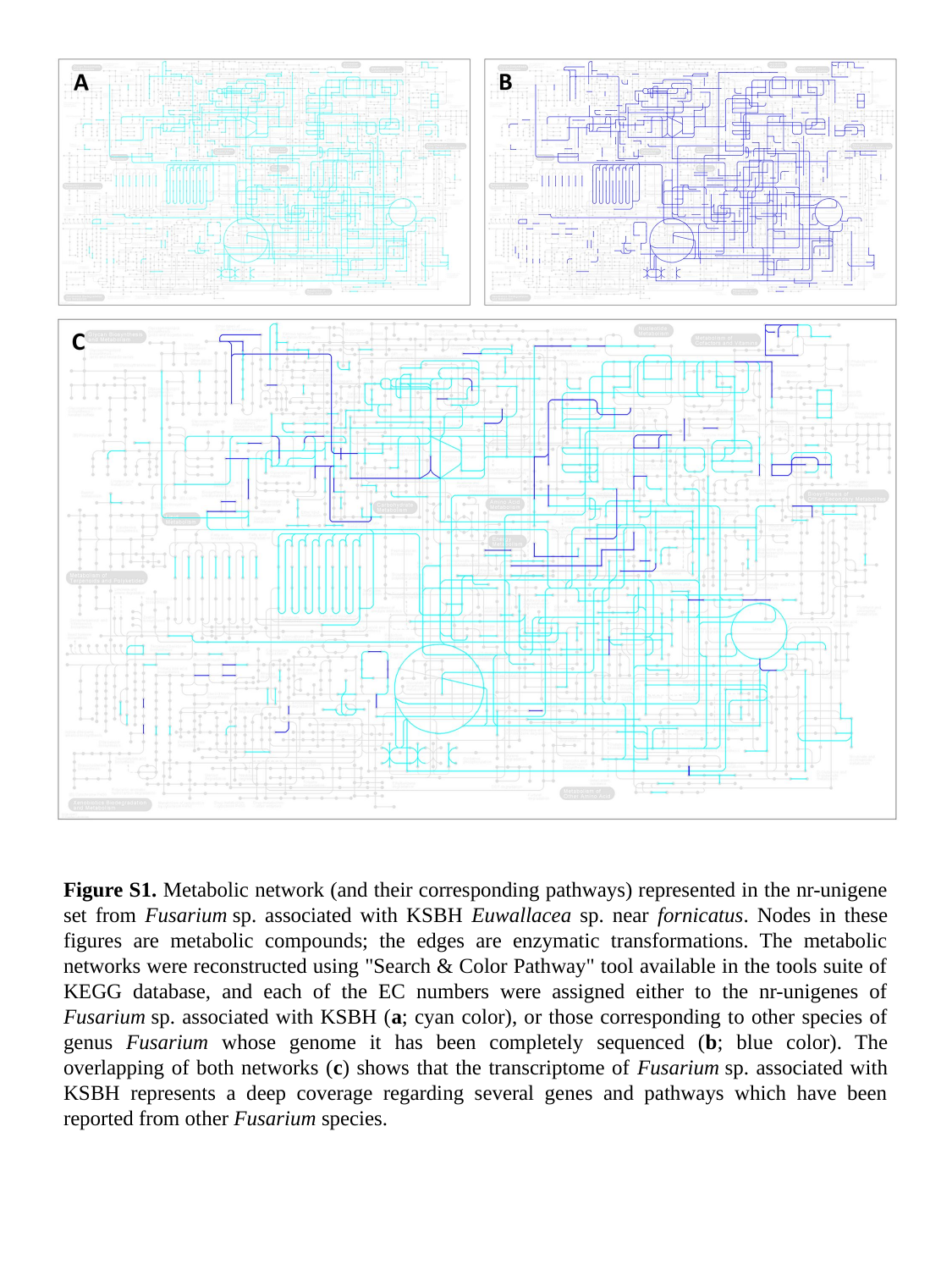

Figure S1. Metabolic network (and their corresponding pathways) represented in the nr-unigene set from Fusarium sp. associated with KSBH Euwallacea sp. near fornicatus. Nodes in these figures are metabolic compounds; the edges are enzymatic transformations. The metabolic networks were reconstructed using "Search & Color Pathway" tool available in the tools suite of KEGG database, and each of the EC numbers were assigned either to the nr-unigenes of Fusarium sp. associated with KSBH (a; cyan color), or those corresponding to other species of genus Fusarium whose genome it has been completely sequenced (b; blue color). The overlapping of both networks (c) shows that the transcriptome of Fusarium sp. associated with KSBH represents a deep coverage regarding several genes and pathways which have been reported from other Fusarium species.

## Slide 2
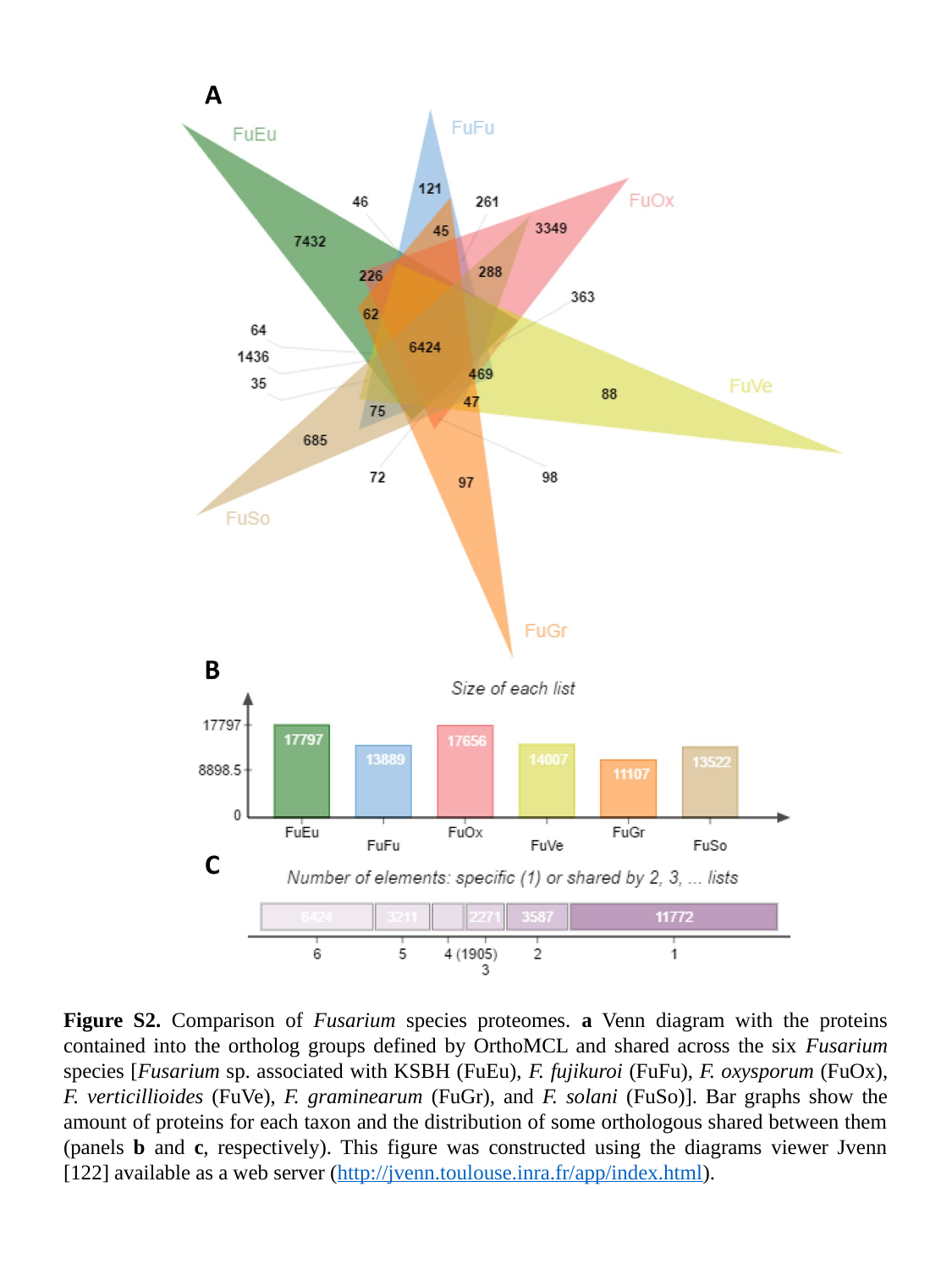

Figure S2. Comparison of Fusarium species proteomes. a Venn diagram with the proteins contained into the ortholog groups defined by OrthoMCL and shared across the six Fusarium species [Fusarium sp. associated with KSBH (FuEu), F. fujikuroi (FuFu), F. oxysporum (FuOx), F. verticillioides (FuVe), F. graminearum (FuGr), and F. solani (FuSo)]. Bar graphs show the amount of proteins for each taxon and the distribution of some orthologous shared between them (panels b and c, respectively). This figure was constructed using the diagrams viewer Jvenn [122] available as a web server (http://jvenn.toulouse.inra.fr/app/index.html).

## Slide 3
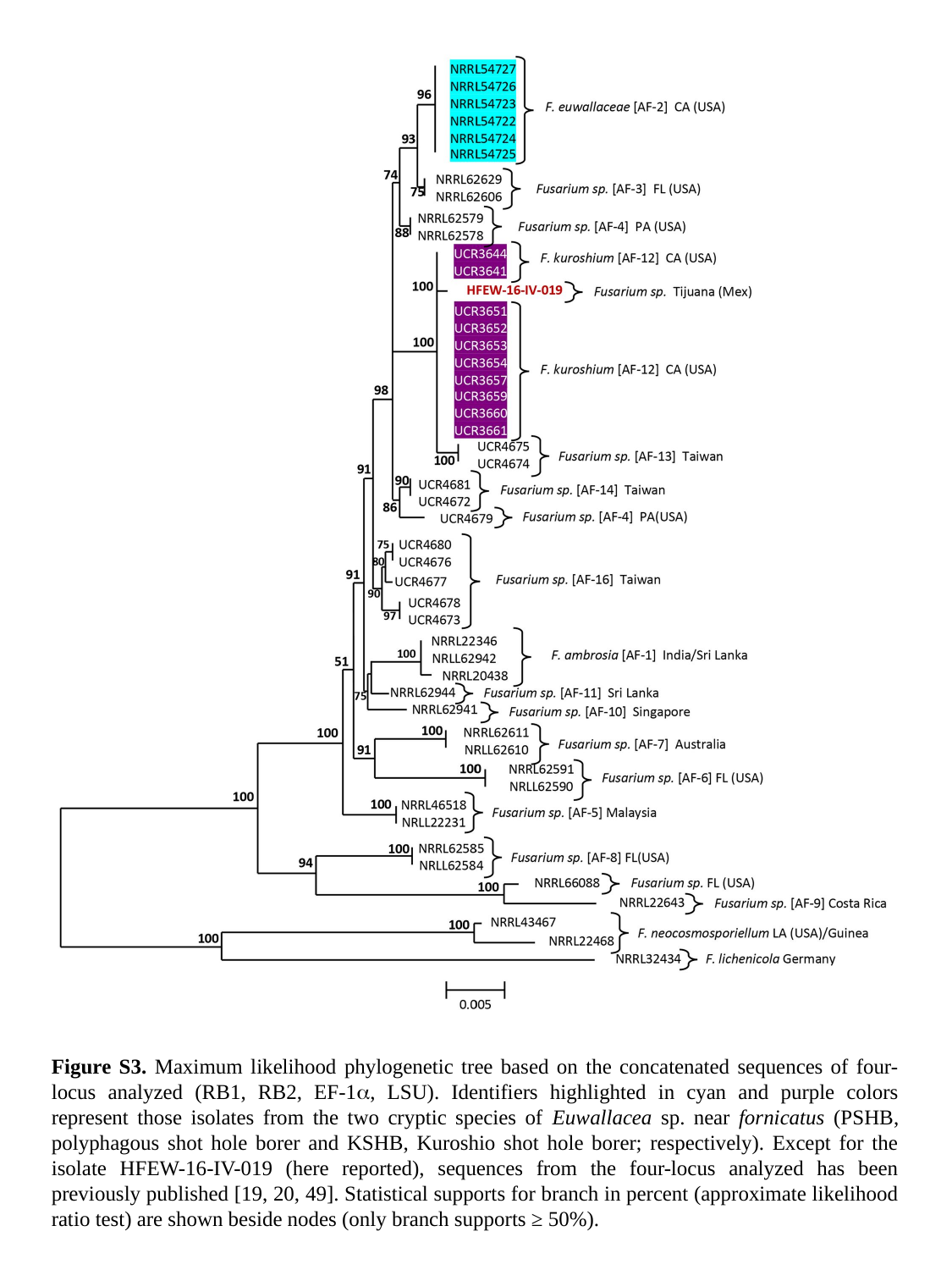

Figure S3. Maximum likelihood phylogenetic tree based on the concatenated sequences of four-locus analyzed (RB1, RB2, EF-1α, LSU). Identifiers highlighted in cyan and purple colors represent those isolates from the two cryptic species of Euwallacea sp. near fornicatus (PSHB, polyphagous shot hole borer and KSHB, Kuroshio shot hole borer; respectively). Except for the isolate HFEW-16-IV-019 (here reported), sequences from the four-locus analyzed has been previously published [19, 20, 49]. Statistical supports for branch in percent (approximate likelihood ratio test) are shown beside nodes (only branch supports ≥ 50%).

## Slide 4
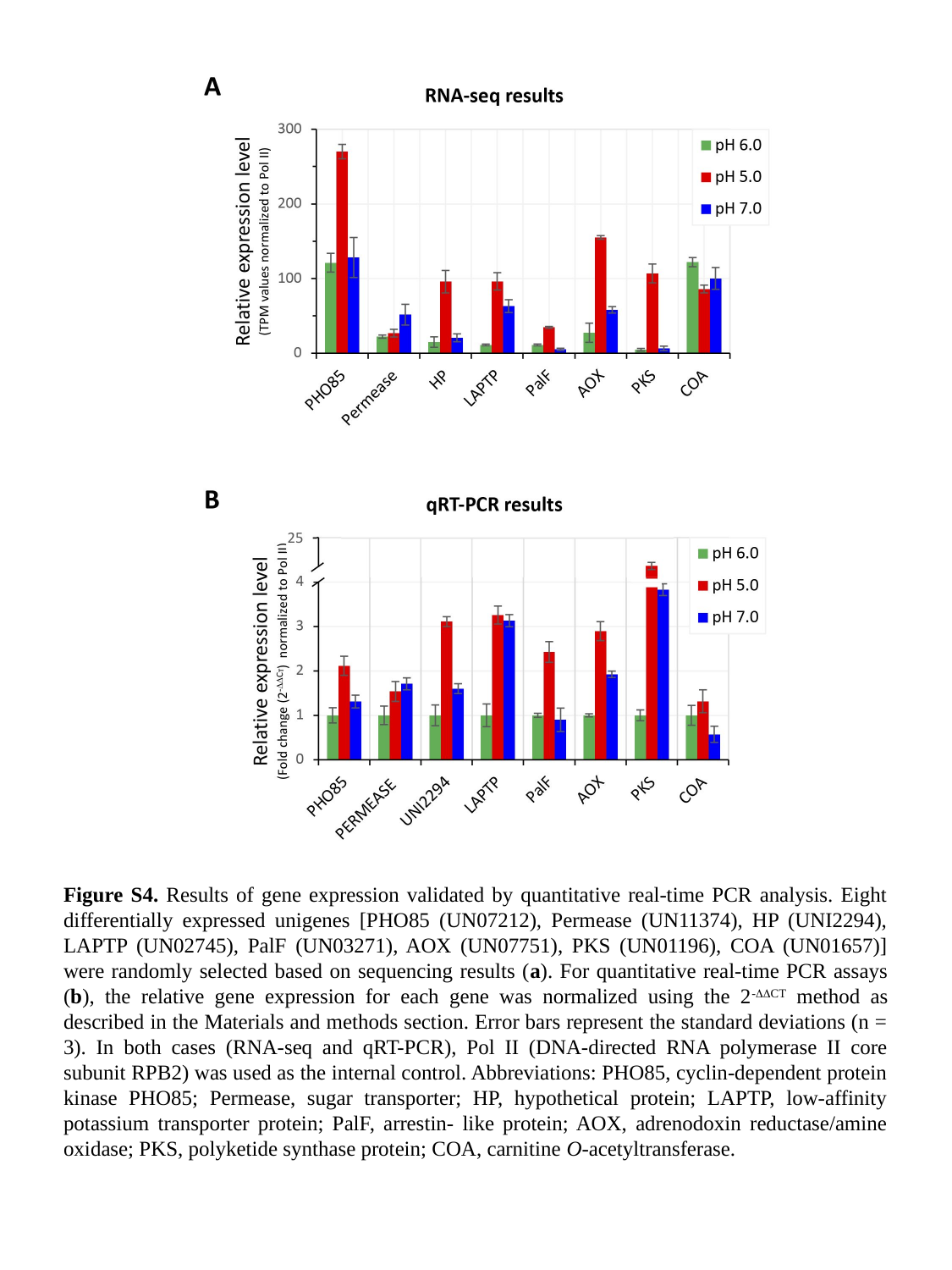

Figure S4. Results of gene expression validated by quantitative real-time PCR analysis. Eight differentially expressed unigenes [PHO85 (UN07212), Permease (UN11374), HP (UNI2294), LAPTP (UN02745), PalF (UN03271), AOX (UN07751), PKS (UN01196), COA (UN01657)] were randomly selected based on sequencing results (a). For quantitative real-time PCR assays (b), the relative gene expression for each gene was normalized using the 2-ΔΔCT method as described in the Materials and methods section. Error bars represent the standard deviations (n = 3). In both cases (RNA-seq and qRT-PCR), Pol II (DNA-directed RNA polymerase II core subunit RPB2) was used as the internal control. Abbreviations: PHO85, cyclin-dependent protein kinase PHO85; Permease, sugar transporter; HP, hypothetical protein; LAPTP, low-affinity potassium transporter protein; PalF, arrestin- like protein; AOX, adrenodoxin reductase/amine oxidase; PKS, polyketide synthase protein; COA, carnitine O-acetyltransferase.

## Slide 5
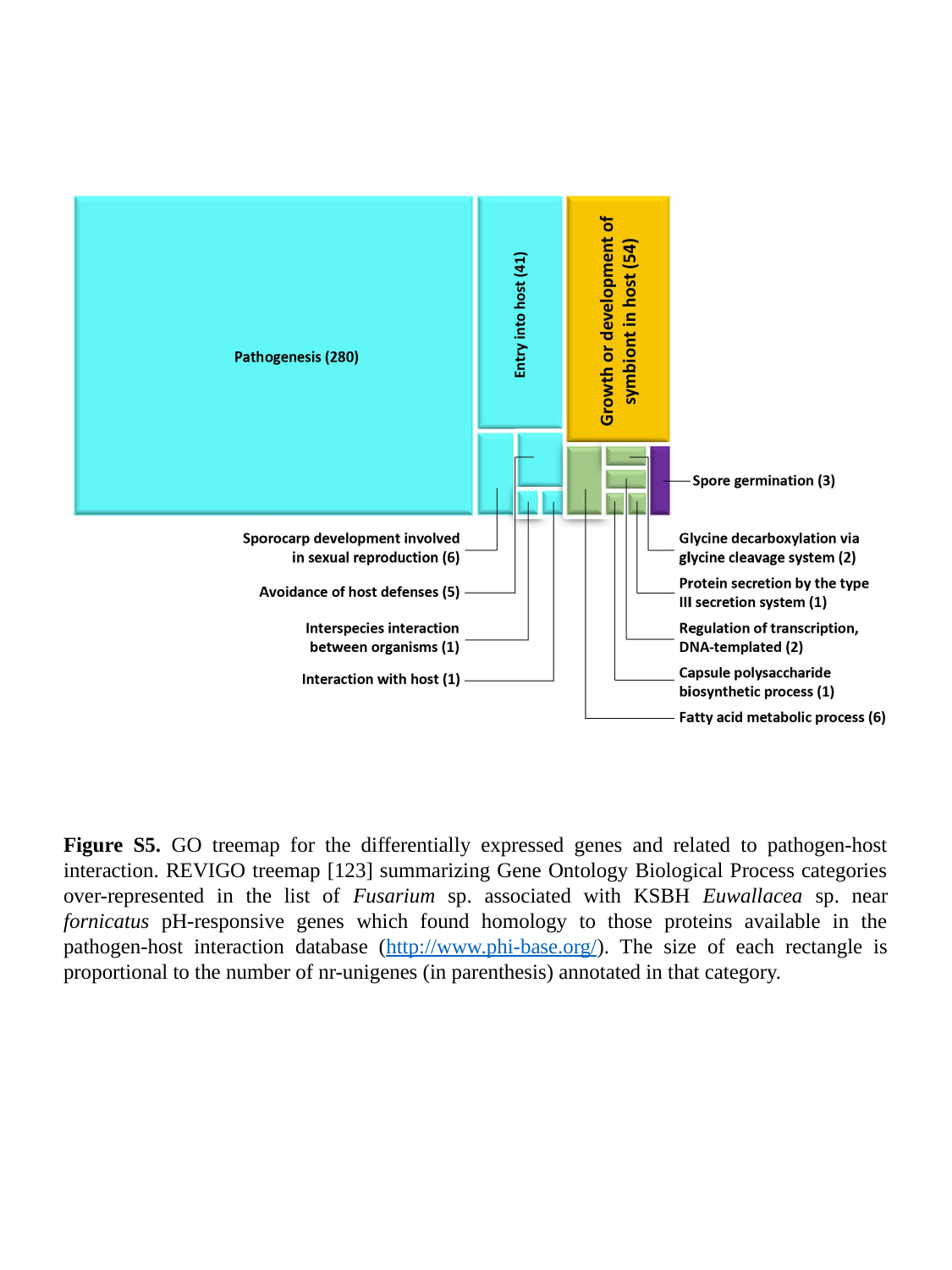

Figure S5. GO treemap for the differentially expressed genes and related to pathogen-host interaction. REVIGO treemap [123] summarizing Gene Ontology Biological Process categories over-represented in the list of Fusarium sp. associated with KSBH Euwallacea sp. near fornicatus pH-responsive genes which found homology to those proteins available in the pathogen-host interaction database (http://www.phi-base.org/). The size of each rectangle is proportional to the number of nr-unigenes (in parenthesis) annotated in that category.

## Slide 6
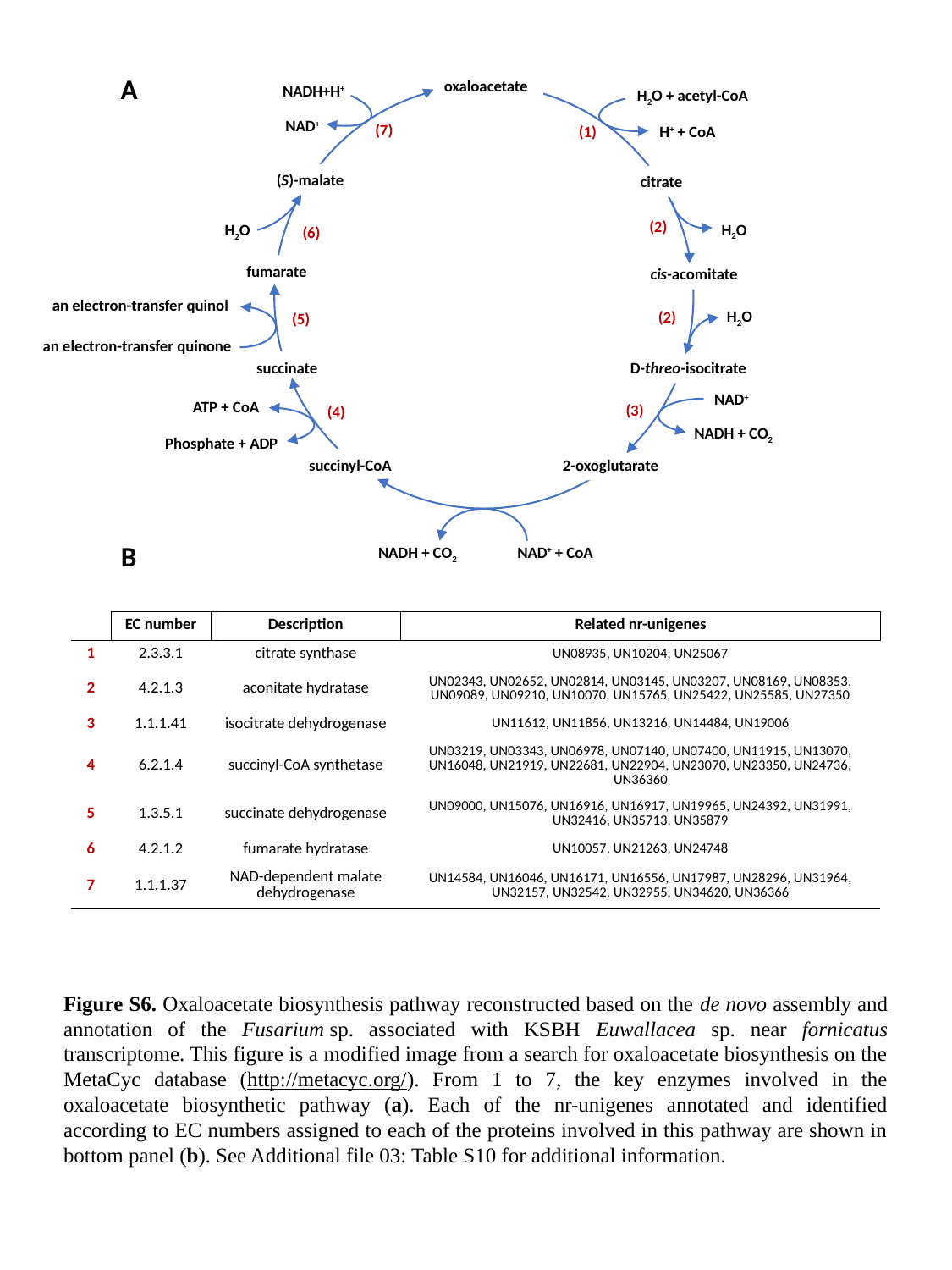

A
oxaloacetate
NADH+H+
H2O + acetyl-CoA
NAD+
(1)
H+ + CoA
(S)-malate
citrate
H2O
H2O
fumarate
cis-acomitate
an electron-transfer quinol
H2O
an electron-transfer quinone
succinate
D-threo-isocitrate
NAD+
ATP + CoA
NADH + CO2
Phosphate + ADP
succinyl-CoA
2-oxoglutarate
NAD+ + CoA
NADH + CO2
(7)
(2)
(6)
(2)
(5)
(3)
(4)
B
| | EC number | Description | Related nr-unigenes |
| --- | --- | --- | --- |
| 1 | 2.3.3.1 | citrate synthase | UN08935, UN10204, UN25067 |
| 2 | 4.2.1.3 | aconitate hydratase | UN02343, UN02652, UN02814, UN03145, UN03207, UN08169, UN08353, UN09089, UN09210, UN10070, UN15765, UN25422, UN25585, UN27350 |
| 3 | 1.1.1.41 | isocitrate dehydrogenase | UN11612, UN11856, UN13216, UN14484, UN19006 |
| 4 | 6.2.1.4 | succinyl-CoA synthetase | UN03219, UN03343, UN06978, UN07140, UN07400, UN11915, UN13070, UN16048, UN21919, UN22681, UN22904, UN23070, UN23350, UN24736, UN36360 |
| 5 | 1.3.5.1 | succinate dehydrogenase | UN09000, UN15076, UN16916, UN16917, UN19965, UN24392, UN31991, UN32416, UN35713, UN35879 |
| 6 | 4.2.1.2 | fumarate hydratase | UN10057, UN21263, UN24748 |
| 7 | 1.1.1.37 | NAD-dependent malate dehydrogenase | UN14584, UN16046, UN16171, UN16556, UN17987, UN28296, UN31964, UN32157, UN32542, UN32955, UN34620, UN36366 |
Figure S6. Oxaloacetate biosynthesis pathway reconstructed based on the de novo assembly and annotation of the Fusarium sp. associated with KSBH Euwallacea sp. near fornicatus transcriptome. This figure is a modified image from a search for oxaloacetate biosynthesis on the MetaCyc database (http://metacyc.org/). From 1 to 7, the key enzymes involved in the oxaloacetate biosynthetic pathway (a). Each of the nr-unigenes annotated and identified according to EC numbers assigned to each of the proteins involved in this pathway are shown in bottom panel (b). See Additional file 03: Table S10 for additional information.

## Slide 7
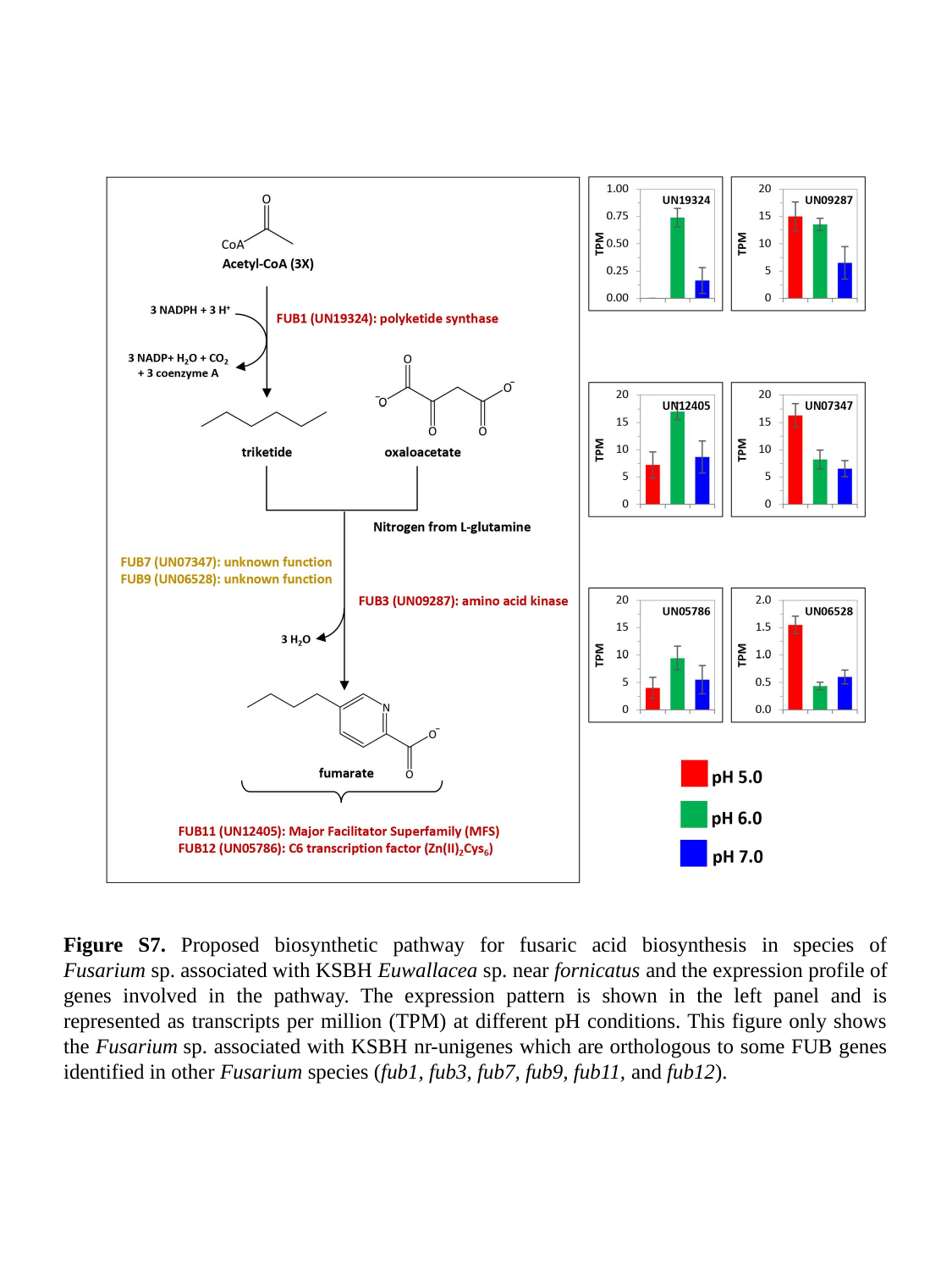

Figure S7. Proposed biosynthetic pathway for fusaric acid biosynthesis in species of Fusarium sp. associated with KSBH Euwallacea sp. near fornicatus and the expression profile of genes involved in the pathway. The expression pattern is shown in the left panel and is represented as transcripts per million (TPM) at different pH conditions. This figure only shows the Fusarium sp. associated with KSBH nr-unigenes which are orthologous to some FUB genes identified in other Fusarium species (fub1, fub3, fub7, fub9, fub11, and fub12).
